# In vivo processing of digital information molecularly with targeted specificity and robust reliability

**DOI:** 10.1126/sciadv.abo7415

**Published:** 2022-08-05

**Authors:** Yangyi Liu, Yubin Ren, Jingjing Li, Fan Wang, Fei Wang, Chao Ma, Dong Chen, Xingyu Jiang, Chunhai Fan, Hongjie Zhang, Kai Liu

**Affiliations:** ^1^ Department of Chemistry, Tsinghua University, Beijing, China.; ^2^ State Key Laboratory of Rare Earth Resource Utilization, Changchun Institute of Applied Chemistry, Chinese Academy of Sciences, Changchun, China.; ^3^ Frontiers Science Center for Transformative Molecules, School of Chemistry and Chemical Engineering, and Institute of Molecular Medicine, Renji Hospital, School of Medicine, Shanghai Jiao Tong University, Shanghai, China.; ^4^ College of Energy Engineering and State Key Laboratory of Fluid Power and Mechatronic Systems, Zhejiang University, Hangzhou, China.; ^5^ Department of Biomedical Engineering, Southern University of Science and Technology, No. 1088 Xueyuan Road, Nanshan District, Shenzhen, Guangdong, China.

## Abstract

DNA has attracted increasing interest as an appealing medium for information storage. However, target-specific rewriting of the digital data stored in intracellular DNA remains a grand challenge because the highly repetitive nature and uneven guanine-cytosine content render the encoded DNA sequences poorly compatible with endogenous ones. In this study, a dual-plasmid system based on gene editing tools was introduced into *Escherichia coli* to process information accurately. Digital data containing large repeat units in binary codes, such as text, codebook, or image, were involved in the realization of target-specific rewriting in vivo, yielding up to 94% rewriting reliability. An optical reporter was introduced as an advanced tool for presenting data processing at the molecular level. Rewritten information was stored stably and amplified over hundreds of generations. Our work demonstrates a digital-to-biological information processing approach for highly efficient data storage, amplification, and rewriting, thus robustly promoting the application of DNA-based information technology.

## Teaser

An in vivo dual-plasmid system was designed for reliable storage, amplification, and rewriting of digital data encoded in DNA.

## INTRODUCTION

As a biological genetic information carrier, deoxyribonucleic acid (DNA) provides a molecular-scale storage option for the vast amount of digital information generated (*1*–*4*). However, the high-precision addressing in DNA sequences has not been achieved via in vitro enzyme reactions, rendering DNA as a medium solely for writing or reading (*5*, *6*). Because of the kaleidoscopic variety of digital data as well as the uneven distribution of bases in information-encoded DNA sequences, target-specific addressing and processing of digital information in living cells is still a great challenge (*7*–*9*). The latest developments in genome editing present tools, which could address target sites, repair damaged sequences, and revise specific genes within living cells (*10*–*13*). Those progresses inspire us to develop innovative biotechnology for in vivo rewriting exogenous information in a versatile way.

CRISPR systems are well known for gene editing in chemical biology (*14*). A variety of CRISPR-associated proteins (Cas), which are guided by their corresponding CRISPR RNA (crRNA) to cleave a target locus in a DNA sequence, appear promising to address and rewrite information-encoded DNA sequences accurately (*15*, *16*). However, the recognition function of Cas is limited by its protospacer-adjacent-motif site, and editing efficiency is severely affected by the secondary structure of guided RNA, rendering the CRISPR-Cas tools highly restricted (*17*–*19*). Thus, arbitrary editing of gene sequences is difficult. In addition, information-encoded DNA sequences record a diverse range of digital information with highly repeating binary codes and have a low compatibility with endogenous DNA sequences, which pose great challenges for the practical applications of CRISPR-Cas tools in DNA-based information storage (*20*). Therefore, target-specific processing of information-encoded DNA sequences within living cells remains unexplored.

In this study, a dual-plasmid system based on a single crRNA-guided endonuclease (CRISPR-Cas12a) was designed and developed for DNA-based information storage and processing in living cells. High storage density, outstanding rewriting reliability, and outstanding amplification stability of digital text, codebook, and image information were successfully demonstrated in vivo using the system. This study explored the application of a dual-plasmid system for DNA-based information storage as well as the specific processing of information-encoded DNA sequences within living cells, such as target-specific rewriting.

## RESULTS

### DNA-based information storage and rewriting within living cells

**Fig. 1.**
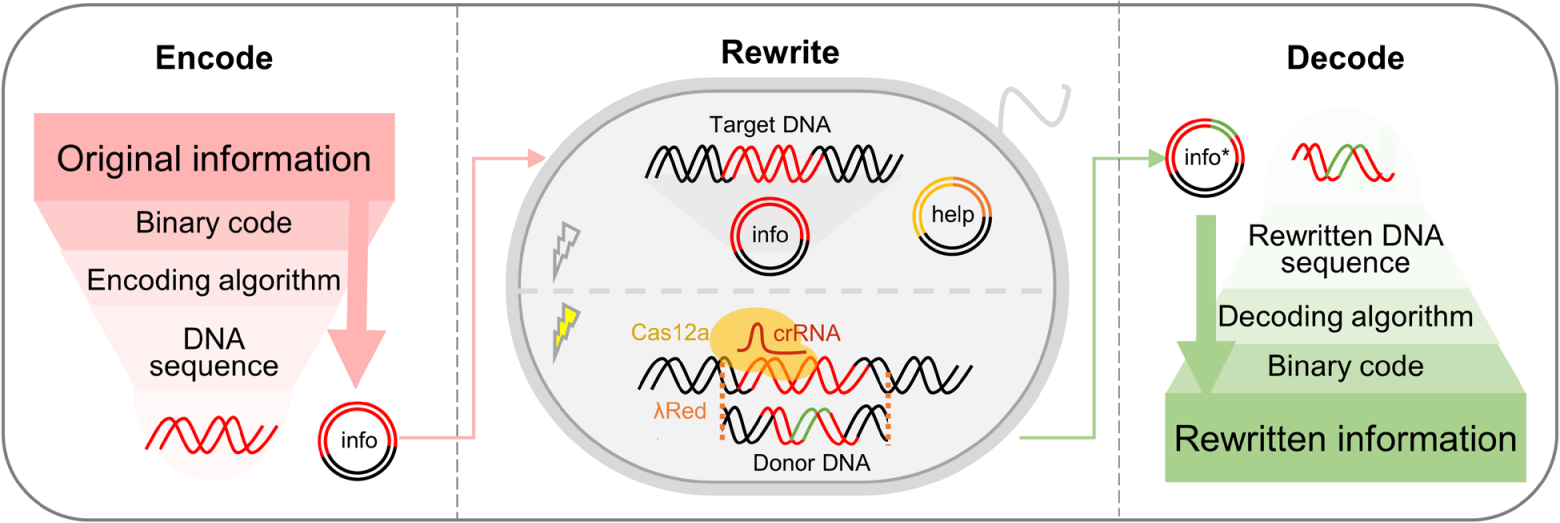
Schematic illustration of DNA-based information storage and rewriting within living cells. The digital information stored in binary codes was encoded into DNA sequences using a high-density encoding algorithm. The sequences were then cloned into the plasmid (info, info plasmid) of living cells for long-term storage, data amplification, and information rewriting. Information rewriting was achieved using the dual-plasmid system based on CRISPR-Cas12a-λRed, whose encoding template was cloned in the plasmid (help, help plasmid). The original information was target-specifically rewritten by replacing the target DNA fragment within the info plasmid with a donor DNA fragment. Following precise revision, the rewritten information was decoded by decoding the DNA sequence within the new info plasmid (info*).

The entire process of DNA-based information storage and rewriting within living cells is schematically illustrated in Fig. 1. The binary codes of the original digital information were first encoded into DNA sequences using a designed high-density encoding algorithm such as Base64 or Huffman (fig. S1) (*21*, *22*). Once the digital binary codes were converted to biological DNA sequences, the information was not easily accessible, and information stored in the DNA sequences could be retrieved only by using the corresponding decoding algorithm. These exogenous information-encoded DNA sequences were then synthesized in vitro by solid-state reaction and cloned in plasmid containing encoded information (info plasmid) of *Escherichia coli* strain MG1655 for long-term storage (*23*).

To rewrite the complex information, a dual-plasmid system based on CRISPR-Cas12a and phage-derived recombinases (λRed) was constructed (*24*). The info plasmid contained the target DNA sequence carrying the original digital information, whereas the other plasmid contained the templates for the expressions of Cas12a and λRed (help plasmid). Following activation of λRed and Cas12a expressions by tetracycline and arabinose, respectively, the target DNA fragment present in the info plasmid was selectively excised by Cas12a under the guidance of crRNA. The donor DNA, which carried the rewritten information and two homologous arms of ~500 base pairs (bp) each, then replaced the target DNA fragment and recombined the info plasmid with the help of λRed (fig. S2). Thus, the original information encoded in the DNA sequence within the info plasmid was selectively revised by replacing the target DNA with donor DNA using the dual-plasmid system.

Following target-specific information rewriting, the rewritten DNA sequence in the info plasmid was sequenced using the universal M13 forward primer (M13F). The precisely revised information was then retrieved by decoding the rewritten DNA sequence using the corresponding decoding algorithm.

### Target-specific rewriting of information encoded by Base64

**Fig. 2.**
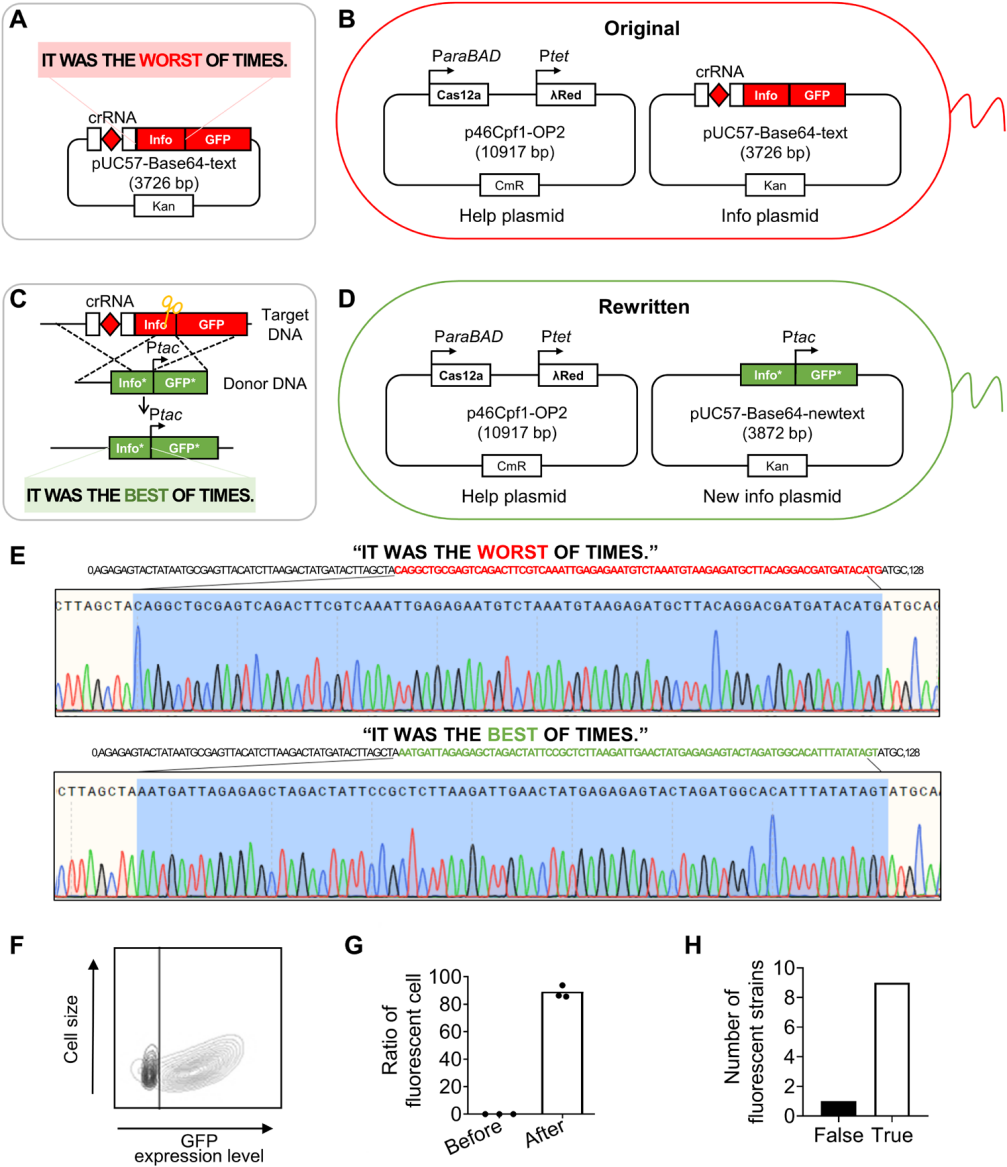
Selective and precise rewriting of information stored in the DNA sequence using the dual-plasmid system. (**A**) The binary codes of a digital text were encoded into a DNA sequence using the Base64 encoding algorithm and synthesized by solid-state reaction. The info plasmid also carried the templates for the constitutive transcription of crRNA and GFP without promoter. (**B**) The info plasmid, pUC57-Base64-text, and the help plasmid, p46Cpf1-OP2, were simultaneously introduced into MG1655 to obtain MG1655BT. The help plasmid expressed λRed recombinases under the control of P*tet* and Cas12a under the control of P*araBAD*. (**C**) Target DNA fragment present in the info plasmid was recognized by crRNA and excised by Cas12a, forming a break in the dsDNA. The donor DNA fragment, which carried the rewritten information, P*tac*, and two ~500-bp homologous arms, replaced the target DNA fragment and recombined the dsDNA assisted by λRed recombinase. (**D**) After rewriting, the original info plasmid was revised into a new info plasmid, pUC57-Base64-newtext. Because the P*tac* promoter was also inserted upstream of the template for GFP (GFP*) expression in the new info plasmid, GFP was successfully expressed in the rewritten strain MG1655BNT, thus serving as a reporter for successful information revision. (**E**) Sequencing of the DNA fragment before and after information rewriting, to retrieve original and rewritten information, respectively. (**F**) GFP expression level in 48-hour-old cells (black contour line: MG1655BT; gray contour line: MG1655BNT) and (**G**) ratio of fluorescent cells before and after information rewriting. (**H**) Successful information rewriting in 9 of 10 fluorescent strains.

To demonstrate the process of information rewriting within living cells, the binary codes of the digital message “IT WAS THE WORST OF TIMES.” from *A Tale of Two Cities* by Charles Dickens were encoded into a 128-bp DNA sequence using the Base64 encoding algorithm (table S1). The Base64 algorithm encodes digital information into DNA sequences with controlled guanine-cytosine (GC) contents and reduced homopolymers, thus reducing the error rates in DNA synthesis and sequencing (*21*). The information-encoded DNA sequence coding by the Base64 algorithm was synthesized by solid-state reaction and stored in the info plasmid, pUC57-Base64-text (table S1). The info plasmid also contained the template for constitutive transcription of crRNA, which recognizes the location of the target DNA fragment for subsequent information revision. In addition, the template for green fluorescence protein (GFP) was cloned without promoter and fused at the 3′ end of information-encoded DNA sequence (Fig. 2A). The help plasmid (p46Cpf1-OP2) expresses λRed recombinases under the control of tetracycline-inducible promoter (P*tet*), as well as Cas12a under the control of the arabinose-inducible promoter (P*araBAD*). The info plasmid and help plasmid were introduced into MG1655 by heat shock and electroporation, respectively, obtaining an *E. coli* strain MG1655BT for long-term storage, data amplification, and information rewriting (Fig. 2B and table S2).

To rewrite the message to “IT WAS THE BEST OF TIMES.” by replacing the word “WORST” with “BEST,” the donor DNA fragment was prepared. The donor DNA, containing 75 bp of rewritten information, a 10-bp hybrid promoter (P*tac*), and two homologous arms of ~500 bp each, was introduced exogenously into MG1655BT by electroporation (Fig. 2C). The two ~500-bp arms were consistent with the upstream and downstream DNA sequences of the target DNA fragment. The target DNA fragment present in the info plasmid was recognized by crRNA. In addition, it was cut by Cas12a, forming a break in the info plasmid. Subsequently, the donor DNA fragment replaced the target DNA fragment and recombined the info plasmid assisted by λRed recombinases. Thus, the original info plasmid was revised to a new info plasmid, pUC57-Base64-newtext (table S1). A rewritten strain MG1655BNT was obtained (Fig. 2D and table S2). After that, to stop rewriting, the strain solution was spread and cultured in the LB medium without the inducer of Cas12a.

In the new info plasmid, the P*tac* promoter was inserted upstream of the template for GFP expression. Because of the trace component of lactose in LB medium, the reporter GFP was successfully expressed in the rewritten strain MG1655BNT. The green fluorescence of reporter GFP enabled the direct visualization and identification of living cells with edited information. To verify the accuracy of the information before and after rewriting, we selected the nonfluorescent strains containing the encoded DNA sequences with original information and the fluorescent strains with the rewritten information. They were verified by sequencing using the universal primer M13F (Fig. 2E). After decoding from the DNA sequences using the Base64 decoding algorithm, both the original and rewritten information were correct, confirming the successful rewriting of fluorescent strains. This was distinguishable by flow cytometry analysis (Fig. 2F). In addition, the ratio of cells showing green fluorescence after rewriting to the total number of cells was up to 89%, suggesting that the rewritten plasmids dominated in most of the cells and the rewriting system exhibited a rather high efficiency regarding data revision (Fig. 2G). To further test the reliability of information rewriting in fluorescent strains after 48 hours of cell culture, we randomly selected 10 fluorescent strains for sequencing. Last, in the nine strains, the info plasmids with the rewriting processing showed the correct information consistently, as summarized in Fig. 2H. Thus, the reliability of information rewriting in fluorescent strains, which is defined as the ratio of fluorescent strains with correct rewritten information to the total number of fluorescent strains, was up to 90%.

The above results suggested that the dual-plasmid system based on CRISPR-Cas12a-λRed was applicable for long-term storage, data amplification, and information rewriting of DNA-based information systems. Furthermore, the information-encoded DNA sequence in the info plasmid was selectively and precisely rewritten within living cells. The successful expression of GFP in the rewritten strains greatly facilitated the identification of strains with rewritten information.

### Selective rewriting of information encoded by 15-ary Huffman

**Fig. 3.**
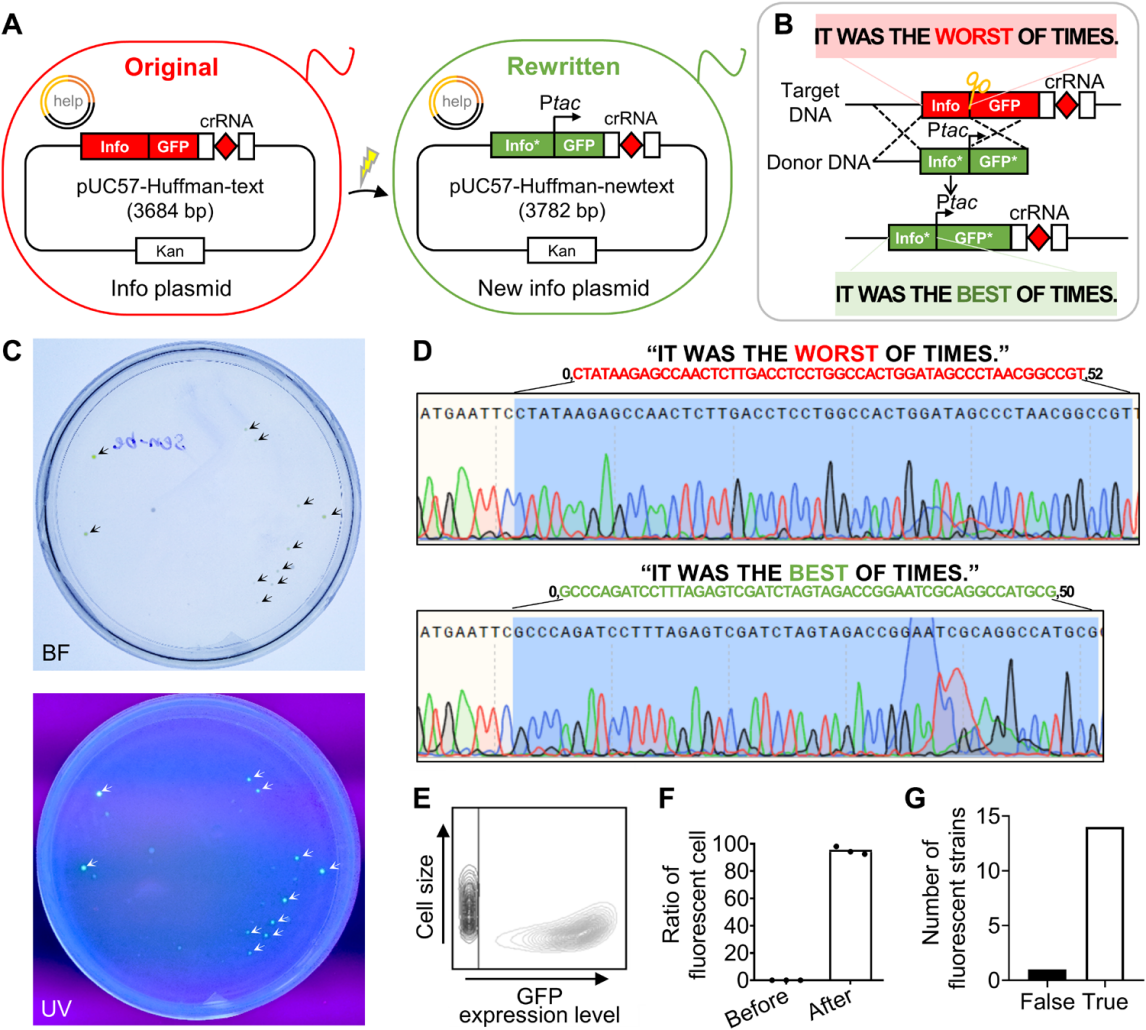
In vivo rewriting of text information encoded by the 15-ary Huffman algorithm. (**A**) The original strain MG1655HT with two plasmids, the info plasmid, pUC57-Huffman-text, and the help plasmid (help), was introduced into *E. coli* MG1655. The rewriting process formed a new info plasmid, pUC57-Huffman-newtext, in the rewritten strain MG1655HNT. (**B**) The binary codes of digital text were encoded into a different DNA sequence using the 15-ary Huffman encoding algorithm, and the synthesized DNA sequence was then stored in the info plasmid of bacteria. The target DNA fragment present in the info plasmid was recognized by crRNA and excised by Cas12a, forming a break in the dsDNA. The donor DNA fragment then replaced the target DNA fragment and recombined the info plasmid assisted by λRed recombinase, thus forming a new info plasmid, pUC57-Huffman-newtext, in the rewritten strain MG1655HNT. (**C**) Green fluorescence of rewritten strains under bright field (BF) and UV light. (**D**) Sequencing of the DNA fragment before and after information rewriting to retrieve original and rewritten information, respectively. (**E**) GFP expression level in 48-hour-old cells (black contour line: MG1655HT; gray contour line: MG1655HNT) and (**F**) ratio of fluorescent cells before and after information rewriting. (**G**) Successful information rewriting in 14 of 15 fluorescent strains.

The Base64 algorithm has the ability to transform binary information into DNA sequences with controlled GC contents and reduced homopolymers. However, for certain information containing large number of repeats, the Base64 algorithm could not reduce the length of the DNA sequence to improve coding efficiency, and homopolymers may still appear. Therefore, the 15-ary Huffman algorithm was developed to compress information by reducing the length of the DNA sequence and avoid the appearance of homopolymers (*22*, *25*). To demonstrate that the dual-plasmid system is also capable of information rewriting using the 15-ary Huffman encoding algorithm, the same text message “IT WAS THE WORST OF TIMES.” was encoded into a 52-bp DNA sequence. The DNA sequence encoded by the 15-ary Huffman encoding algorithm was shorter than that by the Base64 encoding algorithm and contained no homopolymers, as shown in table S1. The word “WORST” was then revised to “BEST” following a similar strategy (Fig. 3, A and B).

The info plasmid encoded text by the 15-ary Huffman algorithm was named pUC57-Huffman-text (table S1). The help plasmid (p46Cpf1-OP2) for rewriting was the same as the former section. Those two plasmids were transferred into MG1655 by heat shock and electroporation, respectively, generating the *E. coli* strain MG1655HT (table S2). To efficiently guide the cleavage of the info plasmid by Cas12a in vitro and in vivo, a 30-bp sequence was designed as the binding site for crRNA (*26*). It was located between the 52-bp information-encoded DNA sequence and the 705-bp sequence of frame code for GFP expression in the info plasmid. The template for the transcription of crRNA was fused at the 3′ end of the 705-bp GFP sequence to easily construct the info plasmid for information rewriting. The target DNA fragment present in the info plasmid was revised in the same way as the previous section, forming a new info plasmid pUC57-Huffman-newtext in the *E. coli* strain MG1655HNT (tables S1 and S2).

The revised DNA sequence within living cells was successfully confirmed by the fluorescent signal of the rewritten strains under ultraviolet (UV) light (Fig. 3C). The information-encoded DNA fragments were then sequenced from nonfluorescent and fluorescent strains, allowing retrieval of original and rewritten information, respectively (Fig. 3D and fig. S6A). Strains were easily distinguished before and after information rewriting by the level of GFP expression (Fig. 3E). After rewriting the information encoded by the 15-ary Huffman encoding algorithm, the ratio of cells showing green fluorescence to the total number of cells was up to 95% (Fig. 3F). In addition, 14 of 15 fluorescent strains showed the correct rewritten information, with the reliability of information rewriting in fluorescent strains being at least 93% (Fig. 3G). Thus, the dual-plasmid system based on CRISPR-Cas12a-λRed is suitable for information rewriting using the 15-ary Huffman algorithm as well.

Compared to the information encoded by the Base64 algorithm, the compression algorithm in the 15-ary Huffman algorithm shortened the information sequence and the donor DNA fragment, improving the coding efficiency to 4.0 bits per nucleotide. However, the compression algorithm increased the difficulty of information rewriting, because small modifications of the original information might result in a large change in the entire DNA sequence, making the 15-ary Huffman algorithm especially suitable for information of small size or those with large repeats. Nevertheless, homopolymers were completely eliminated in the DNA sequence encoded by the 15-ary Huffman algorithm, which greatly reduced the error rates in DNA synthesis and sequencing.

**Fig. 4.**
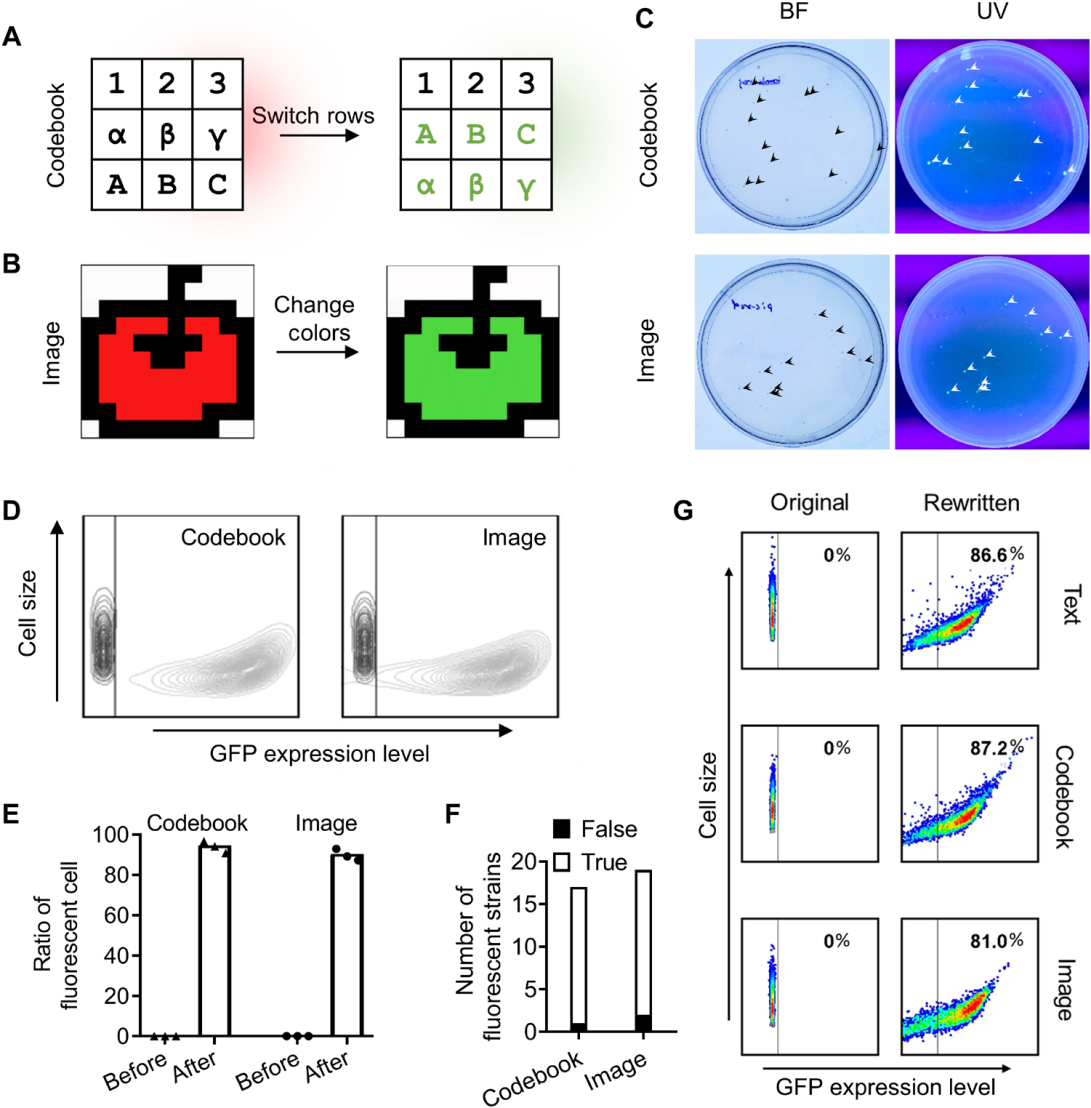
Rewriting of codebook and image stored in the DNA sequences using CRISPR-Cas12a-λRed. (**A**) Schematics showing row switch in the codebook and (**B**) color change in the image, achieved by information rewriting. (**C**) Green fluorescent color of rewritten strains under UV light, which confirmed the revision of codebook and image encoded in the DNA sequences using the dual-plasmid system based on CRISPR-Cas12a-λRed. (**D**) GFP expression level in 48-hour-old cells (black contour line: MG1655HC and MG1655HI; gray contour line: MG1655HNC and MG1655HNI) and (**E**) ratio of fluorescent cells before and after information rewriting. (**F**) Successful row switch of codebook information in 17 of 18 fluorescent strains and successful color change of image information in 19 of 21 fluorescent strains. (**G**) Flow cytometry analysis of cell size and GFP expression level in 48-hour-old cells of unrevised strains (MG1655HT, MG1655HC, and MG1655HI) and rewritten strains (MG1655HNT, MG1655HNC, and MG1655HNI) after the fifth inoculation. The high GFP expression level observed after the fifth inoculation suggests that the rewritten information can be passed on to future generations.

Generally, it is difficult to store digital information with large repeat units in binary codes in DNA sequences, such as codebook and image. They are hard to synthesize and recognize by crRNA for rewriting within living cells. To address this challenge, a codebook with a size of 56 bytes, which contained nine characters in a 3 × 3 matrix, was converted into a 140-bp DNA sequence with a coding efficiency of 3.3 bits per nucleotide by the 15-ary Huffman encoding algorithm. The second row in the codebook was successfully switched with the third following the same strategy as that used for text information rewriting, as schematically illustrated in Fig. 4A. The information-encoded DNA fragments were sequenced from nonfluorescent and fluorescent strains, which carry the original and revised information, respectively (fig. S6B). The original and revised information decoded from the DNA sequences are 100% correct.

Furthermore, we tested an image, which is notably larger than the codebook. The 376-byte large image was encoded into a 748-bp sequence with a coding efficiency of 4.0 bits per nucleotide by the 15-ary Huffman encoding algorithm. After rewriting the DNA sequence, the color of the apple pattern changed from red to green, as illustrated in Fig. 4B. The revised information was further confirmed by DNA sequencing (fig. S6C).

In the case of codebook and image information, the expression of GFP and the resulting green fluorescence in the rewritten strains under UV light served as a good reporter for successful information rewriting (Fig. 4, C and D). The ratio of cells showing green fluorescence to the total number of rewritten cells was up to 94% for the codebook and 90% for the image (Fig. 4E); the reliability of information rewriting in fluorescent strains reached 94% for the codebook and 91% for the image (Fig. 4F). The reliability of information rewriting for the image was slightly lower as compared to that for the codebook, which can be attributed to the large size of the image. Owing to its advanced algorithm and the explored crRNA sequence, the capability of the dual-plasmid system to precisely rewrite the exogenous information sequence is comparable to that of the existing gene editing system (*27*). Notably, this dual-plasmid system based on the prokaryotic genome editing tool, CRISPR-Cas12a-λRed, proved to be a feasible strategy for long-term storage, amplification, and rewriting of different types of information containing large repeat units in binary codes, including text, codebook, and image.

### Stability of DNA-based information stored in living cells

*E. coli* bacteria generally proliferate into the next generation within 20 min under optimal conditions. Mutations during plasmid replication or changes in cellular fitness may compromise the fidelity of DNA-based information stored within living cells (*28*). To test the genetic stability of the information, *E. coli* strains were randomly chosen and inoculated consecutively five times. MG1655HT, MG1655HC, and MG1655HI, storing three different types of information encoded by the 15-ary Huffman encoding algorithm, were cultured. Following information revision, the rewritten fluorescent strains MG1655HNT, MG1655HNC, and MG1655HNI were reinoculated every 12 hours at 30°C, during which bacteria were allowed to proliferate under optimal culture conditions (*29*). After the fifth inoculation, the bacteria were cultured for 36 hours at 30°C and showed a high GFP expression level as detected by flow cytometry (Fig. 4G and fig. S7). During the whole process, bacteria were cultured for a total of 84 hours, and one bacterium could proliferate over 252 generations into 7.2 × 10^75^ bacteria. The copy of information stored in the bacteria was exponentially amplified simultaneously. Notably, the decoded DNA fragment was sequenced from the 252nd generation of the fluorescent strains. The results indicated that the rewritten information was 100% correct, suggesting that the DNA-based information stored in bacteria was robust, and the expression of GFP has little effect on the stability of the DNA sequence (fig. S8). Furthermore, the genomic DNA of the information-encoded *E. coli* strains before and after rewriting was studied by next-generation sequencing. The average rate of single-nucleotide variants among the rewritten information-encoded strains was only 0.000030, which was much lower than the control group (fig. S9). This confirmed that there was almost no off-target and mutant behaviors in the process of rewriting and storing. Thus, the DNA-based information stored in bacteria could be passed on to future generations, providing a versatile and reliable way to amplify the information.

## DISCUSSION

We developed an in vivo dual-plasmid system using a rational design of coding algorithm and an information editing tool. It is suitable and universal for storing, rewriting, and reading various types of information, including text, codebook, and image. The system fully explores the coding capability of DNA sequences without requiring any addressing indices or backup sequences and is compatible with various kinds of coding algorithms, thus enabling a high coding efficiency. Notably, the coding efficiency of DNA-based information was achieved by incorporating compression algorithm with rotation mapping in the 15-ary Huffman algorithm. This aimed to reduce the length of the encoded DNA sequence for some information containing a large number of repeats and to avoid the appearance of homopolymers. Eventually, the efficiency was up to 4.0 bits per nucleotide in the present systems, which can be an alternative strategy to improve coding efficiency (*6*, *7*, *13*, *21*, *30*–*40*), as shown in fig. S10. In addition, the digital information encoded in info plasmids can be stored and hidden in microbial colonies stably over hundreds of generations for a long duration of time, greatly improving the security of information during storage and transportation.

To achieve efficiency as well as reliability in the rewriting of the complex information stored in exogenous DNA sequences in vivo, the high specificity between complementary pairs of nucleic acid molecules was used to accurately construct new information. By optimizing the crRNA sequence, the information rewriting tool became highly adaptable to complex information, resulting in a high rewriting reliability of up to 94% and comparable to that of existing gene editing systems (*27*, *41*). In addition, because of the Cas12a/crRNA complex being activated by exogenous inducers, the rewriting process could be maneuvered to initiate at a desired time and space (*42*). The introduction of fluorescent proteins as reporters also greatly improved the readability of living cells carrying rewritten information and resulted in the direct visibility of the molecular-level editing of DNA sequences. Hence, this study realized the application of the CRISPR-Cas tool for the processing and revision of various types of digital information within living cells. By combining the CRISPR-Cas tool and the coding algorithm, the dual-plasmid system presented as a universal platform for DNA-based information rewriting in vivo, thus depicting a new strategy for information processing and target-specific rewriting of large and complicated data on a molecular level.

In summary, digital information stored in exogenous DNA sequences was specifically targeted and reliably rewritten within living cells using the dual-plasmid CRISPR-Cas12a system. The digital-to-biological system enables DNA, a molecular-level information carrier, to be processed as traditional physical memory with targeted specific access and information editing. The system also serves as a novel paradigm for flexible and dynamic processing of digital information within living cells. While this dual-plasmid system is low cost and nimble, the storage capacity is limited by the host genome size and the length of inserted DNA. Future exploration of the system in a living host with a larger genome and adoption of plasmids such as yeast artificial chromosome, which allows the insertions of longer artificial DNA sequences, would further pave the way for practical applications regarding big data storage. Furthermore, this system could combine with multiple functional elements, such as silica biomineralization peptides and light-inducible transcription factors for long-term data storage and adjustable information processing, respectively. The system could also integrate with microfluidic technology to build a living cell information storage array (*43*). Nevertheless, this study widens the platform of DNA-based information storage and highlights the infinite potential applications of chemical biology in multiple disciplines.

## MATERIALS AND METHODS

### Materials

Propagation, subcloning, and storage of plasmids were performed within *E. coli* DH5α purchased from TransGen (China). Recombinant proteins were expressed in *E. coli* BL21(DE3) purchased from TransGen (China). *E. coli* MG1655 purchased from ZOMANBIO (China) were used for information storage and rewriting. DNA sequences including info plasmid, new information sequences, and the template for GFP expression (705 bp) were synthesized by GENEWIZ (China) and are listed in table S1. The strains used in this study are listed in table S2. Primers in table S3 were synthesized by Tsingke (China). After DNA amplification, procedures including DNA cloning, DNA fragment fusion, and polymerase chain reaction (PCR) test were performed to construct the donor DNA fragment, which used the enzymes purchased from Vazyme (China). DNA purification and plasmid isolation kits were purchased from TIANGEN (China).

### Construction of the dual-plasmid system

The dual-plasmid system was constructed by first introducing 100 ng of info plasmid into 50 μl of chemocompetent MG1655 cells via heat shock. The resulting cells were incubated at 37°C for 1 hour and were spread on 1.5% agarose plate with kanamycin (50 μg/ml) to isolate the strains that contain the info plasmid. The plate was cultured at 37°C for 12 hours, and one isolated strain was cultured in 1 ml of LB medium with kanamycin (50 μg/ml) at 30°C under 220 rpm vibration overnight. After centrifugation at 4000*g* for 2 min at 4°C, collected cells were washed two times with 1 ml of ice-cold 10 vol% glycerol solution, suspended in 200 μl of ice-cold 10 vol% glycerol solution, and separated into 40 μl per tube. One hundred nanograms of help plasmid was then introduced into 40 μl of MG1655 cells containing info plasmid and incubated on ice for 1 min. The cells were then transferred to a chilled electroporation cuvette with a gap of 2 mm (Bio-Rad, USA) and pulsed once at 2.5 kV for 2.5 ms with a Bio-Rad GenePulser (Bio-Rad, USA). After electroporation using MG1655, cells carrying both info and help plasmids were incubated with kanamycin (50 μg/ml) and chloramphenicol (12.5 μg/ml) at 30°C.

### Construction of the donor DNA

The DNA fragment carrying the rewritten information was synthesized by solid-state reaction. To construct the donor DNA, two ~500-bp homologous arms, which are consistent with the upstream and downstream DNA sequences of the target DNA fragment, were fused with the DNA fragment at its upstream and downstream, respectively. The homologous arms were amplified from info plasmid by a pair of primers. For text information encoded by the Base64 algorithm, the upstream homologous arm was amplified by Ba-rewrite-F and Ba-UParm-R from the upstream DNA sequence of the target DNA fragment in the info plasmid, and the downstream homologous arm was amplified by Ba-DWarm-F and Ba-rewrite-R from the downstream DNA sequence of the target DNA fragment. For information encoded by the 15-ary Huffman algorithm, the upstream homologous arm was amplified by Hu-rewrite-F and Hu-UParm-R from the upstream DNA sequence of the target DNA fragment in the info plasmid, and the downstream homologous arm was amplified by Hu-DWarm-F and Hu-rewrite-R from the downstream DNA sequence of the target DNA fragment. Before information rewriting, there is no promoter for the expression of GFP. Promoter P*tac*, which was contained in the donor DNA, was fused at the 5′ end of template for GFP expression after information rewriting, and thus, GFP was successfully expressed in rewritten strains, which serves as a good reporter for rewritten strains.

### Functions of crRNA, Cas12a, λRed, and donor DNA in the dual-plasmid system

The cutting of info plasmids by Cas12a under the guidance of crRNA was tested in vitro. The selected cutting site of the info plasmid pUC57-Base64-text was designed in the information sequence. However, for the info plasmid pUC57-Huffman-text, the selected cutting site recognized by crRNA was fused with the 3′ end of the information sequence. To synthesize the cutting guider crRNA in vitro, DNA templates were prepared, which contained a T7 promoter, 25-nt guide sequence, and a crRNA scaffold. The templates were generated by annealing a pair of primers, such as Ba-crRNA-F and Ba-crRNA-R for info plasmid pUC57-Base64-text or Hu-crRNA-F and Hu-crRNA-R for info plasmid pUC57-Huffman-text. The pair of primers are dissolved in 20 μl of sterile water at a concentration of 1 μM. The annealing process included heating at 95°C for 5 min and cooling from 95° to 4°C at a rate of −1°C/min. The annealed double-stranded DNA (dsDNA) templates were then transcribed and purified in vitro using the T7 High-Yield RNA Transcription Kit and VAHTS RNA Clean Beads purchased from Vazyme (China). For the expression of Cas12a, the plasmid pET-28b-T7-henAsCas12a-HF1 was purchased from Addgene (America, #114073) and introduced into *E. coli* BL21(DE3). Subsequently, recombinant Cas12a was expressed by *E. coli* and purified by His-tag column (fig. S3A). Then, cutting of info plasmid by the purified Cas12a in vitro was performed according to the manufacturer’s instruction (*44*). Briefly, the in vitro reaction containing info plasmid (12 ng/μl), Cas12a protein (50 ng/μl), and crRNA (35 ng/μl) in 10 μl of solution (containing 20 mM Hepes, 150 mM KCl, 1 mM MgCl_2_, and 10% glycerol) was performed at 37°C for 1 hour. The sample was then treated with 4 μg of ribonuclease purchased from Vazyme (China) and incubated at 37°C for 15 min; subsequently, the sample was treated with 2.5 μg of proteinase K purchased from Sigma-Aldrich (USA) and incubated at 55°C for 10 min. After 5 min at room temperature, the sample received 1 μl of STOP solution (30% glycerol, 1.2% SDS, and 250 mM EDTA at pH 8.0) and was incubated at 37°C for 15 min. Last, the sample was analyzed by gel electrophoresis in 1% agarose gel (fig. S3B).

*E. coli* MG1655 bearing the dual-plasmid system were incubated with kanamycin (50 μg/ml) and chloramphenicol (12.5 μg/ml) at 30°C. In the living cells, crRNA was constitutively transcribed under the control of P*speI*. λRed recombinase, which was expressed under the control of P*tet*, was promoted at the addition of tetracycline (80 ng/ml). In the presence of l-arabinose (5 mg/ml) and the absence of glucose (20 mg/ml), Cas12a was expressed and cut the target DNA sequence in the info plasmid under the guidance of crRNA. Rewritten information was encoded in the donor DNA sequence, and 2 μl of purified donor DNA (100 ng/μl) was introduced into the dual-plasmid system. The info plasmid with a donor DNA sequence aims to accomplish information rewriting. Therefore, the functions of crRNA, Cas12a, λRed, and donor DNA in the dual-plasmid system were tested in the crRNA-free, Cas12a-free, λRed-free, and donor DNA–free groups, respectively, by turning off each individual induction signal and incubating the system at 30°C for 1 day. The results are shown in fig. S4.

### Information rewriting using the dual-plasmid system

A schematic diagram illustrating the process for information rewriting using the dual-plasmid system is shown in fig. S2. The procedure is described in detail below: (i) Day 1: Individual colony containing info plasmid and help plasmid was picked and grew in LB medium with kanamycin (50 μg/ml), chloramphenicol (12.5 μg/ml), and glucose (20 mg/ml) at 30°C under 220 rpm vibration overnight. (ii) Day 2: 10 μl of the above bacterial culture was added into 1 ml of LB medium with kanamycin (50 μg/ml) and chloramphenicol (12.5 μg/ml) in a 1.5-ml eppendorf (EP) tube and then cultured at 30°C under 220 rpm vibration for 6 hours. l-Arabinose (5 mg/ml) and tetracycline (80 ng/ml) were then added into the bacterial culture. After incubation at 30°C for 2 hours, the sample was centrifuged at 4000*g* for 2 min at 4°C. The cells were washed two times with 1 ml of ice-cold 10 vol% glycerol solution, then suspended in 200 μl of ice-cold 10 vol% glycerol solution, and separated into 40 μl per tube. Two hundred nanograms of donor DNA was added into 40 μl of resuspended cells for electroporation. The electroporation condition was the same as Construction of the dual-plasmid system. The cells after electroporation were resuspended by 1 ml of Super Optimal broth with Catabolite repression (SOC) medium and transferred into a 1.5-ml EP tube. After incubation at 30°C for 1 hour, l-arabinose (5 mg/ml) and tetracycline (80 ng/ml) were added into the bacterial culture and incubated for another 1 hour. The bacterial culture was then centrifuged at 4000*g* for 5 min at room temperature, and collected cells were redispersed in 100 μl of LB medium, which were spread on 1.5% agarose plate with kanamycin (50 μg/ml), chloramphenicol (12.5 μg/ml), and glucose (20 mg/ml) and cultured at 30°C for 48 hours. (iii) Days 3 to 4: Individual strain was observed under UV light to distinguish green fluorescent strains, which were then sequenced by the first-generation sequencing method using the universal primer M13F.

### Flow cytometry analysis of fluorescent strains

After information rewriting and incubation for 48 hours, all strains on the plate were harvested and suspended in 1 ml of PBS in a 1.5-ml EP tube. The cells were washed two times with 1 ml of PBS and resuspended in 1 ml of PBS. The cell suspension was filtered through a stainless steel mesh (300 mesh), and the green fluorescence attributed to the expression of GFP was detected by the flow cytometer. Blank control consisted of strains before information rewriting, which were also harvested from 1.5% agarose plate with kanamycin (50 μg/ml), chloramphenicol (12.5 μg/ml), and glucose (20 mg/ml).

### Next-generation sequencing of the information-encoded strains

After information rewriting and incubation for 48 hours, the *E. coli* strains MG1655HT, MG1655HNT, MG1655HC, MG1655HNC, MG1655HI, and MG1655HNI were harvested and suspended in 200 μl of 20 vol% glycerin in PCR tube, which was stored at 4°C. The negative control was the MG1655 strain with p46Cpf1-OP2, which was cultured at 30°C overnight and stimulated by l-arabinose (5 mg/ml) at 30°C for 24 hours. Each strain was inoculated in 5 ml of LB media, proliferated at 30°C for 20 hours, and collected by centrifugation for sequencing (GENEWIZ, China). For next-generation sequencing, each sample was sequenced on the Illumina HiSeq X Ten/NovaSeq/MGISEQ-2000 System. The annotation for single-nucleotide variants was performed by Annovar (V21 April 2018) and referred the sequence of MG1655 complete genome (National Center for Biotechnology Information reference sequence: NC_000913.3, https://ncbi.nlm.nih.gov/nuccore/556503834?report=fasta).

## Supplementary Materials

### This PDF file includes:

Figs. S1 to S10 Tables S1 to S3 References

## Notes

View/request a protocol for this paper from *Bio-protocol*.

## References

[1] Y. Hao, Q. Li, C. Fan, F. Wang, Data storage based on DNA. Small Struct. 2, 2000046 (2021).

[2] D. Bennet, T. Vo-Dinh, F. Zenhausern, Current and emerging opportunities in biological medium-based computing and digital data storage. Nano Select 3, 883–902 (2022).

[3] K. Matange, J. M. Tuck, A. J. Keung, DNA stability: A central design consideration for DNA data storage systems. Nat. Commun. 12, 1358 (2021). 33649304 10.1038/s41467-021-21587-5 PMC7921107

[4] B. H. Nguyen, C. N. Takahashi, G. Gupta, J. A. Smith, R. Rouse, P. Berndt, S. Yekhanin, D. P. Ward, S. D. Ang, P. Garvan, H.-Y. Parker, R. Carlson, D. Carmean, L. Ceze, K. Strauss, Scaling DNA data storage with nanoscale electrode wells. Sci. Adv. 7, eabi6714 (2021). 34818035 10.1126/sciadv.abi6714 PMC8612674

[5] L. C. Meiser, P. L. Antkowiak, J. Koch, W. D. Chen, A. X. Kohll, W. J. Stark, R. Heckel, R. N. Grass, Reading and writing digital data in DNA. Nat. Protoc. 15, 86–101 (2020). 31784718 10.1038/s41596-019-0244-5

[6] J. Koch, S. Gantenbein, K. Masania, W. J. Stark, Y. Erlich, R. N. Grass, A DNA-of-things storage architecture to create materials with embedded memory. Nat. Biotechnol. 38, 39–43 (2020). 31819259 10.1038/s41587-019-0356-z

[7] L. Organick, S. D. Ang, Y. J. Chen, R. Lopez, S. Yekhanin, K. Makarychev, M. Z. Racz, G. Kamath, P. Gopalan, B. Nguyen, C. N. Takahashi, S. Newman, H. Y. Parker, C. Rashtchian, K. Stewart, G. Gupta, R. Carlson, J. Mulligan, D. Carmean, G. Seelig, L. Ceze, K. Strauss, Random access in large-scale DNA data storage. Nat. Biotechnol. 36, 242–248 (2018). 29457795 10.1038/nbt.4079

[8] L. Organick, Y. J. Chen, S. Dumas Ang, R. Lopez, X. Liu, K. Strauss, L. Ceze, Probing the physical limits of reliable DNA data retrieval. Nat. Commun. 11, 616 (2020). 32001691 10.1038/s41467-020-14319-8 PMC6992699

[9] J. L. Banal, T. R. Shepherd, J. Berleant, H. Huang, M. Reyes, C. M. Ackerman, P. C. Blainey, M. Bathe, Random access DNA memory using Boolean search in an archival file storage system. Nat. Mater. 20, 1272–1280 (2021). 34112975 10.1038/s41563-021-01021-3 PMC8564878

[10] W. Tang, D. R. Liu, Rewritable multi-event analog recording in bacterial and mammalian cells. Science 360, eaap8992 (2018). 29449507 10.1126/science.aap8992 PMC5898985

[11] H. Lee, D. J. Wiegand, K. Griswold, S. Punthambaker, H. Chun, R. E. Kohman, G. M. Church, Photon-directed multiplexed enzymatic DNA synthesis for molecular digital data storage. Nat. Commun. 11, 5246 (2020). 33067441 10.1038/s41467-020-18681-5 PMC7567835

[12] S. S. Yim, R. M. McBee, A. M. Song, Y. Huang, R. U. Sheth, H. H. Wang, Robust direct digital-to-biological data storage in living cells. Nat. Chem. Biol. 17, 246–253 (2021). 33432236 10.1038/s41589-020-00711-4 PMC7904632

[13] W. Chen, M. Han, J. Zhou, Q. Ge, P. Wang, X. Zhang, S. Zhu, L. Song, Y. Yuan, An artificial chromosome for data storage. Natl. Sci. Rev. 8, nwab028 (2021). 34691648 10.1093/nsr/nwab028 PMC8288405

[14] C. Arnold, What’s new in clinical CRISPR? Nat. Med. 27, 184–185 (2021). 33510440 10.1038/s41591-020-01222-4

[15] R. Brosh, J. M. Laurent, R. Ordonez, E. Huang, M. S. Hogan, A. M. Hitchcock, L. A. Mitchell, S. Pinglay, J. A. Cadley, R. D. Luther, D. M. Truong, J. D. Boeke, M. T. Maurano, A versatile platform for locus-scale genome rewriting and verification. Proc. Natl. Acad. Sci. U.S.A. 118, e2023952118 (2021). 33649239 10.1073/pnas.2023952118 PMC7958457

[16] Z. Wu, Y. Zhang, H. Yu, D. Pan, Y. Wang, Y. Wang, F. Li, C. Liu, H. Nan, W. Chen, Q. Ji, Programmed genome editing by a miniature CRISPR-Cas12f nuclease. Nat. Chem. Biol. 17, 1132–1138 (2021). 34475565 10.1038/s41589-021-00868-6

[17] J. Champer, J. Liu, S. Y. Oh, R. Reeves, A. Luthra, N. Oakes, A. G. Clark, P. W. Messer, Reducing resistance allele formation in CRISPR gene drive. Proc. Natl. Acad. Sci. U.S.A. 115, 5522–5527 (2018). 10.1073/pnas.1720354115 PMC6003519 29735716

[18] A. V. Anzalone, P. B. Randolph, J. R. Davis, A. A. Sousa, L. W. Koblan, J. M. Levy, P. J. Chen, C. Wilson, G. A. Newby, A. Raguram, D. R. Liu, Search-and-replace genome editing without double-strand breaks or donor DNA. Nature 576, 149–157 (2019). 31634902 10.1038/s41586-019-1711-4 PMC6907074

[19] M. Naeem, S. Majeed, M. Z. Hoque, I. Ahmad, Latest developed strategies to minimize the off-target effects in CRISPR-Cas-mediated genome editing. Cell 9, 1608 (2020). 10.3390/cells9071608 PMC7407193 32630835

[20] S. L. Shipman, J. Nivala, J. D. Macklis, G. M. Church, CRISPR–Cas encoding of a digital movie into the genomes of a population of living bacteria. Nature 547, 345–349 (2017). 28700573 10.1038/nature23017 PMC5842791

[21] Y. Zhang, L. Kong, F. Wang, B. Li, C. Ma, D. Chen, K. Liu, C. Fan, H. Zhang, Information stored in nanoscale: Encoding data in a single DNA strand with Base64. Nano Today 33, 100871 (2020).

[22] Y. Ren, Y. Zhang, Y. Liu, Q. Wu, J. Su, F. Wang, D. Chen, C. Fan, K. Liu, H. Zhang, DNA-based concatenated encoding system for high-reliability and high-density data storage. Small Methods 6, e2101335 (2022). 35146964 10.1002/smtd.202101335

[23] D. Choe, J. H. Lee, M. Yoo, S. Hwang, B. H. Sung, S. Cho, B. Palsson, S. C. Kim, B.-K. Cho, Adaptive laboratory evolution of a genome-reduced Escherichia coli. Nat. Commun. 10, 935 (2019). 30804335 10.1038/s41467-019-08888-6 PMC6389913

[24] X. Ao, Y. Yao, T. Li, T. T. Yang, X. Dong, Z. T. Zheng, G. Q. Chen, Q. Wu, Y. Guo, A multiplex genome editing method for Escherichia coli based on CRISPR-Cas12a. Front. Microbiol. 9, 2307 (2018). 30356638 10.3389/fmicb.2018.02307 PMC6189296

[25] Y. Ren, Y. Zhang, Y. Liu, Q. Wu, H.-G. Hu, J. Li, C. Fan, D. Chen, K. Liu, H. Zhang, Highly reliable and efficient encoding systems for hexadecimal polypeptide-based data storage. Fundam. Res. 10.1016/j.fmre.2021.11.030, (2021). 10.1016/j.fmre.2021.11.030 PMC11197718 38932929

[26] J. S. Chen, E. Ma, L. B. Harrington, M. D. Costa, X. Tian, J. M. Palefsky, J. A. Doudna, CRISPR-Cas12a target binding unleashes indiscriminate single-stranded DNase activity. Science 360, 436–439 (2018). 29449511 10.1126/science.aar6245 PMC6628903

[27] L. Zhang, R. Sun, M. Yang, S. Peng, Y. Cheng, C. Chen, Conformational dynamics and cleavage sites of Cas12a are modulated by complementarity between crRNA and DNA. iScience 19, 492–503 (2019). 31437752 10.1016/j.isci.2019.08.005 PMC6710298

[28] M. Hao, H. Qiao, Y. Gao, Z. Wang, X. Qiao, X. Chen, H. Qi, A mixed culture of bacterial cells enables an economic DNA storage on a large scale. Commun. Biol. 3, 416 (2020). 32737399 10.1038/s42003-020-01141-7 PMC7395121

[29] P. Wang, L. Robert, J. Pelletier, W. L. Dang, F. Taddei, A. Wright, S. Jun, Robust growth of *Escherichia coli*. Curr. Biol. 20, 1099–1103 (2010). 20537537 10.1016/j.cub.2010.04.045 PMC2902570

[30] G. M. Church, Y. Gao, S. Kosuri, Next-generation digital information storage in DNA. Science 337, 1628 (2012). 22903519 10.1126/science.1226355

[31] N. Goldman, P. Bertone, S. Chen, C. Dessimoz, E. M. LeProust, B. Sipos, E. Birney, Towards practical, high-capacity, low-maintenance information storage in synthesized DNA. Nature 494, 77–80 (2013). 23354052 10.1038/nature11875 PMC3672958

[32] S. M. Yazdi, Y. Yuan, J. Ma, H. Zhao, O. Milenkovic, A rewritable, random-access DNA-based storage system. Sci. Rep. 5, 14138 (2015). 26382652 10.1038/srep14138 PMC4585656

[33] R. N. Grass, R. Heckel, M. Puddu, Robust chemical preservation of digital information on DNA in silica with error-correcting codes. Angew. Chem. Int. Ed. 54, 2552–2555 (2015). 10.1002/anie.201411378 25650567

[34] J. Bornholt, R. Lopez, D. M. Carmean, L. Ceze, G. Seelig, K. Strauss, paper presented at the Proceedings of the 21st International Conference on Architectural Support for Programming Languages and Operating Systems, Atlanta, GA, USA, April 2016, pp. 2–6.

[35] C. Mayer, G. R. McInroy, P. Murat, P. Van Delft, S. Balasubramanian, An epigenetics-inspired DNA-based data storage system. Angew. Chem. Int. Ed. 55, 11144–11148 (2016). 10.1002/anie.201605531 PMC5113786 27440712

[36] R. Lopez, Y. J. Chen, S. Dumas Ang, S. Yekhanin, K. Makarychev, M. Z. Racz, G. Seelig, K. Strauss, L. Ceze, DNA assembly for nanopore data storage readout. Nat. Commun. 10, 2933 (2019). 31270330 10.1038/s41467-019-10978-4 PMC6610119

[37] L. Anavy, I. Vaknin, O. Atar, R. Amit, Z. Yakhini, Data storage in DNA with fewer synthesis cycles using composite DNA letters. Nat. Biotechnol. 37, 1229–1236 (2019). 31501560 10.1038/s41587-019-0240-x

[38] H. H. Lee, R. Kalhor, N. Goela, J. Bolot, G. M. Church, Terminator-free template-independent enzymatic DNA synthesis for digital information storage. Nat. Commun. 10, 2383 (2019). 31160595 10.1038/s41467-019-10258-1 PMC6546792

[39] Y. Choi, T. Ryu, A. C. Lee, H. Choi, H. Lee, J. Park, S. H. Song, S. Kim, H. Kim, W. Park, S. Kwon, High information capacity DNA-based data storage with augmented encoding characters using degenerate bases. Sci. Rep. 9, 6582 (2019). 31036920 10.1038/s41598-019-43105-w PMC6488701

[40] K. J. Tomek, K. Volkel, E. W. Indermaur, J. M. Tuck, A. J. Keung, Promiscuous molecules for smarter file operations in DNA-based data storage. Nat. Commun. 12, 3518 (2021). 34112775 10.1038/s41467-021-23669-w PMC8192770

[41] C. Xu, B. Ma, Z. Gao, X. Dong, C. Zhao, H. Liu, Electrochemical DNA synthesis and sequencing on a single electrode with scalability for integrated data storage. Sci. Adv. 7, eabk0100 (2021). 34767438 10.1126/sciadv.abk0100 PMC8589306

[42] Y. Chen, Y. Mei, X. Jiang, Universal and high-fidelity DNA single nucleotide polymorphism detection based on a CRISPR/Cas12a biochip. Chem. Sci. 12, 4455–4462 (2021). 34163711 10.1039/d0sc05717g PMC8179484

[43] J. Sun, J. Chen, K. Liu, H. Zeng, Mechanically strong proteinaceous fibers: Engineered fabrication by microfluidics. Engineering 7, 615–623 (2021).

[44] N. Kalebic, E. Taverna, S. Tavano, F. K. Wong, D. Suchold, S. Winkler, W. B. Huttner, M. Sarov, CRISPR/Cas9-induced disruption of gene expression in mouse embryonic brain and single neural stem cells in vivo. EMBO Rep. 17, 338–348 (2016). 26758805 10.15252/embr.201541715 PMC4772980

[45] S. L. Shipman, J. Nivala, J. D. Macklis, G. M. Church, Molecular recordings by directed CRISPR spacer acquisition. Science 353, aaf1175 (2016). 27284167 10.1126/science.aaf1175 PMC4994893

[46] Y. Mei-Yi, Y. Hai-Qin, R. Gai-Xian, Z. Ju-Ping, G. Xiao-Peng, S. Yi-Cheng, CRISPR-Cas12a-assisted recombineering in bacteria. Appl. Environ. Microbiol. 83, e00947-17 (2017). 28646112 10.1128/AEM.00947-17 PMC5561284

[47] H. A. De Boer, L. J. Comstock, M. Vasser, The *tac* promoter: A functional hybrid derived from the *trp* and *lac* promoters. Proc. Natl. Acad. Sci. U.S.A. 80, 21–25 (1983). 6337371 10.1073/pnas.80.1.21 PMC393301

