# Supplementary material for: In vivo processing of digital information molecularly with targeted specificity and robust reliability
Source: Sci Adv. 2022 Aug 5;8(31):eabo7415. doi: 10.1126/sciadv.abo7415 (PMC9355361; doi:10.1126/sciadv.abo7415)
Supplement: Supplementary file 1 — Figs. S1 to S10 Tables S1 to S3 References [file sciadv.abo7415_sm.pdf]

Science Advances
AAAS

Supplementary Materials for

In vivo processing of digital information molecularly with targeted specificity and robust reliability

Yangyi Liu *et al.*

Corresponding author: Jingjing Li, jjingli@ciac.ac.cn; Dong Chen, chen_dong@zju.edu.cn;
Kai Liu, kailiu@tsinghua.edu.cn

Sci. Adv. **8**, eabo7415 (2022)
DOI: 10.1126/sciadv.abo7415

This PDF file includes:

Figs. S1 to S10
Tables S1 to S3
References

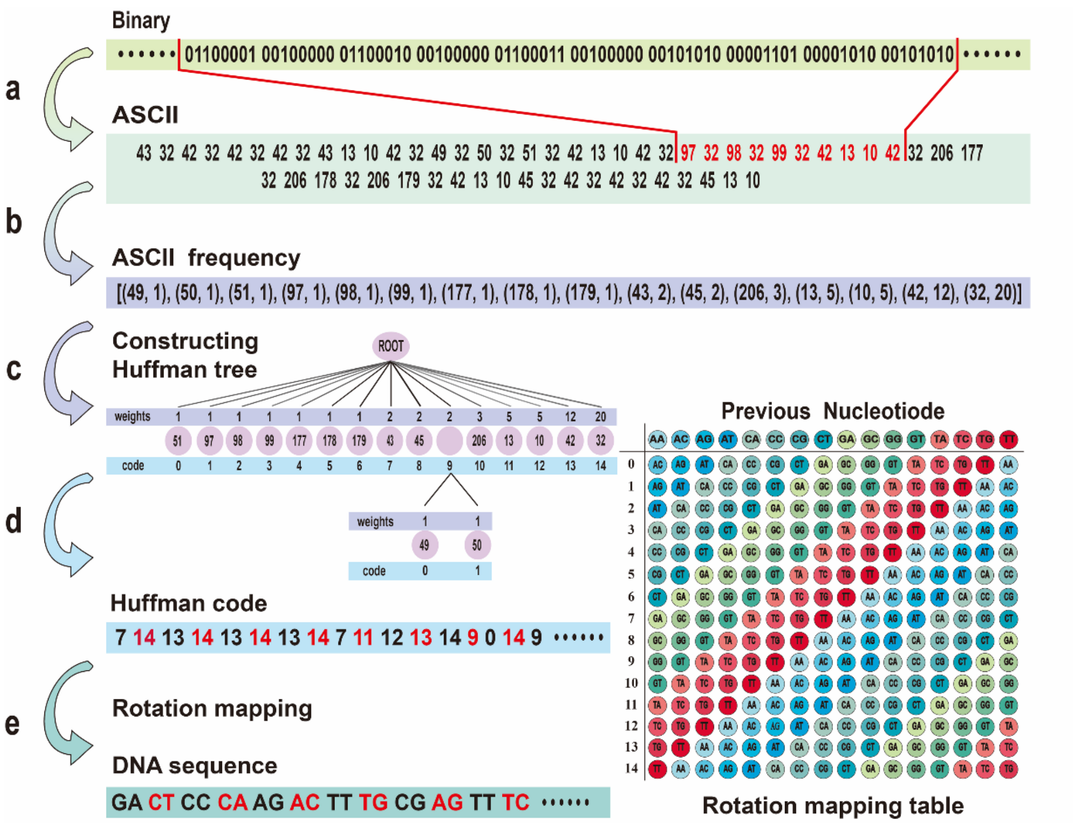

Fig. S1. 15-ary Huffman algorithm for high-density information storage in DNA. (A) The binary sequence was divided into 8-bit units and each 8-bit unit was converted into a corresponding ASCII code. **(B)** The frequency of each ASCII code was counted and then the ASCII codes were sorted according to their frequency. **(C)** and **(D)** A 15-ary Huffman tree was constructed to convert the ASCII codes into Huffman codes within the range of [0,14]. **(E)** The Huffman codes were converted into DNA sequences according to the rotation mapping table.

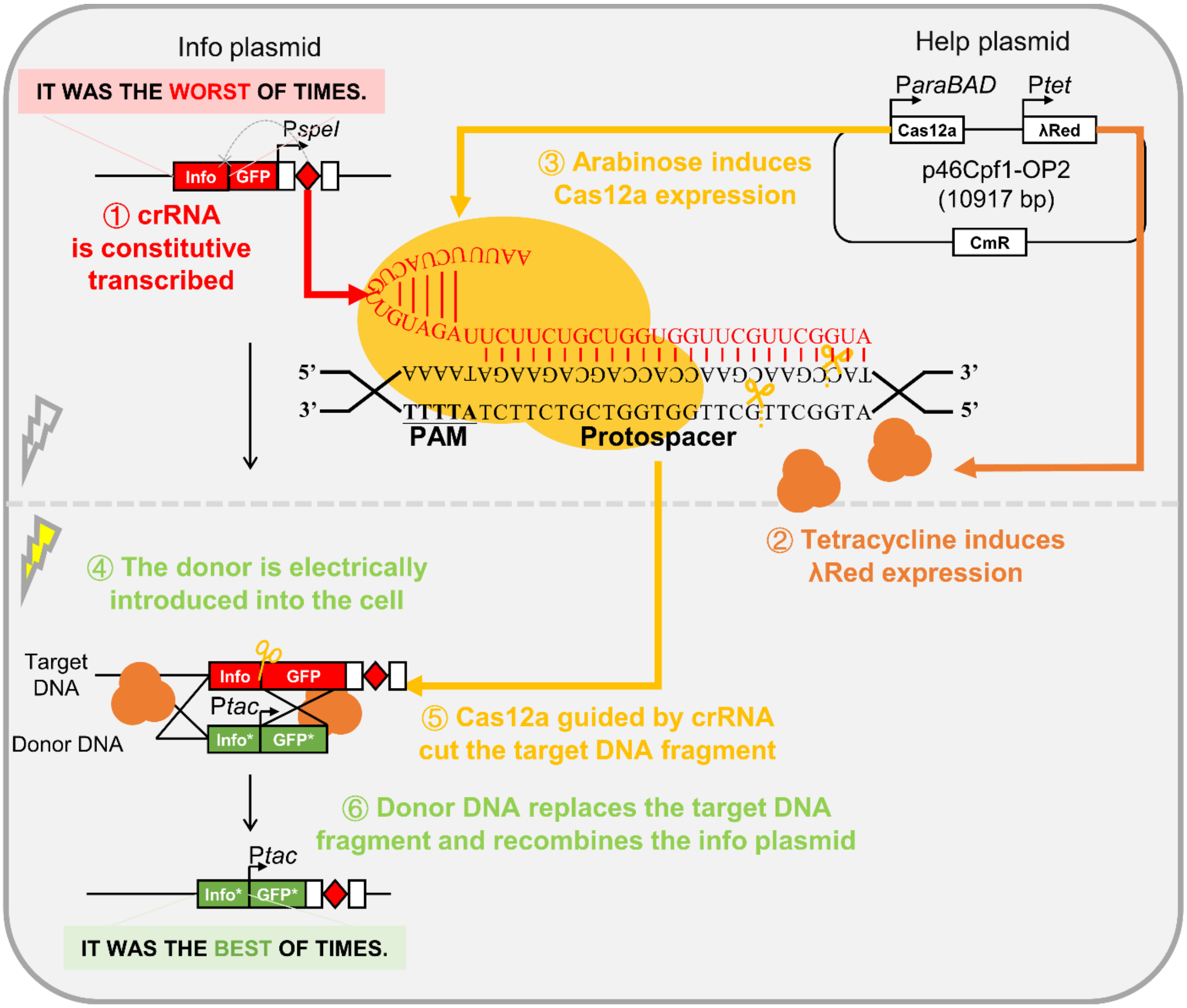

Fig. S2. Process of information rewriting using the two-plasmid system based on CRISPR-Cas12a-λRed. In the two-plasmid system, crRNA, which locates the cutting site, was constitutively transcribed from the info plasmid under the control of P*speI* (red arrow). After the addition of tetracycline, λRed was expressed from the help plasmid under the control of P*tet* (orange arrow). To initiate information rewriting, arabinose was introduced into the culture medium, which promotes P*araBAD* to express Cas12a. Guided by crRNA, Cas12a found the target PAM site (TTTTA) and cleave the target DNA fragment in the info plasmid, inducing a break (yellow arrow). Donor DNA with two 500 bp homologous arms was then introduced into the cell by electroporation. Subsequently, donor DNA replaces the target DNA fragment and recombines the info plasmid assisted by λRed within the living cell.

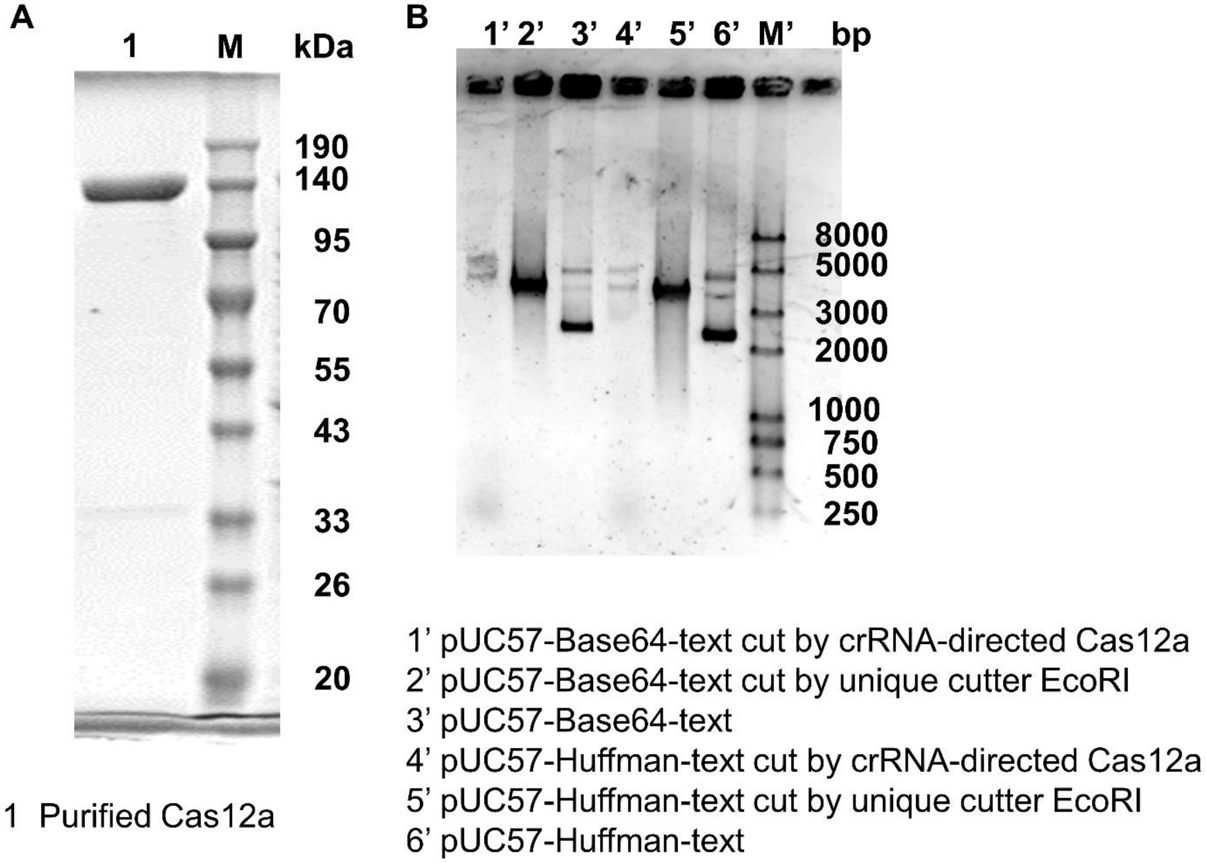

Fig. S3. Molecular weight of info plasmids investigated by gel electrophoresis. The molecular weights of info plasmids under different treatments match with theoretical prediction, suggesting the successful cleavage of info plasmids by the CRISPR-Cas12 system. **(A)** SDS-PAGE gel electrophoresis of Cas12a expressed by *E. coli* and purified by His-tag column as shown in Lane 1. Protein size markers are shown in Lane M. **(B)** Agarose gel electrophoresis of info plasmids cut by crRNA-directed Cas12a (Lanes 1’ and 4’), info plasmids cut by unique cutter EcoRI (Lanes 2’ and 5’), info plasmids before cutting (Lanes 3’ and 6’) and DNA size markers (Lane M’). 1 mg purified Cas12a and 500 ng crRNA, which were transcribed *in vitro*, were used to cut the info plasmids (*44*).

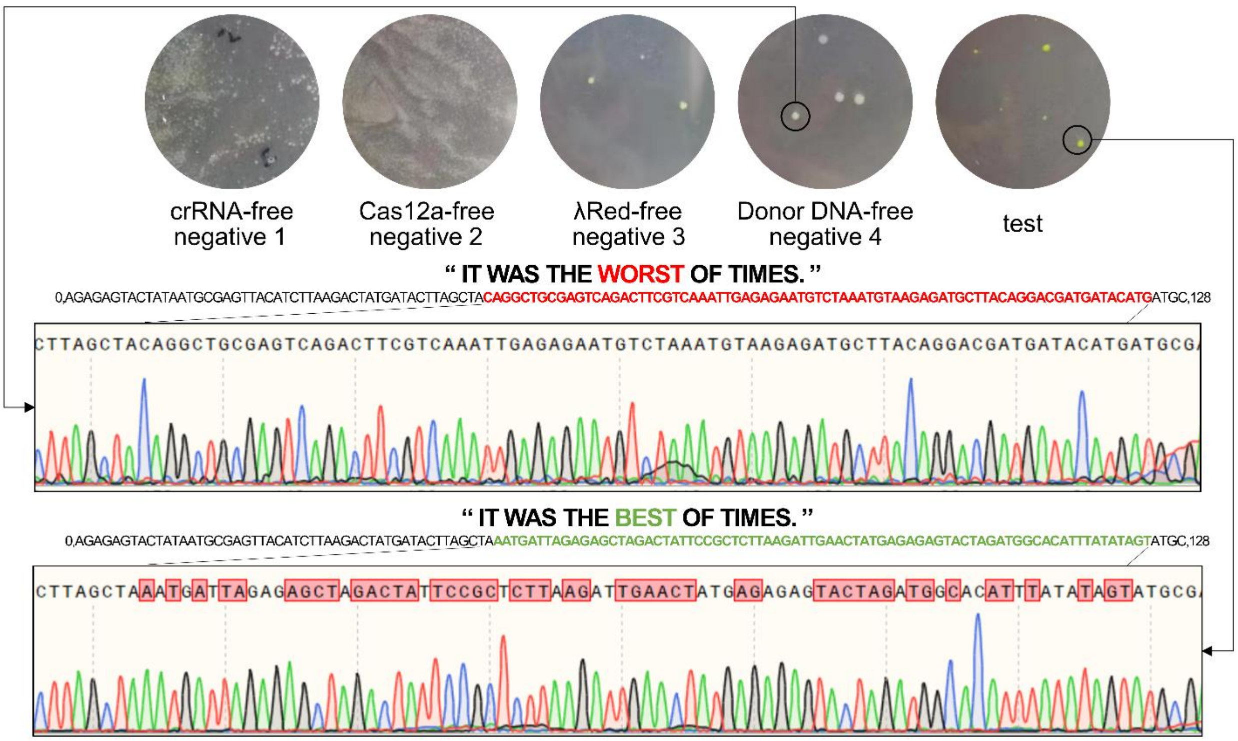

Fig. S4. Important roles of crRNA, Cas12a, λRed and donor DNA for information rewriting. In crRNA-free groups, Cas12a could not cleave the info plasmid without the guidance of crRNA. In Cas12a-free groups, the info plasmid could not be cleaved. In λRed-free groups, the break cut by Cas12a in the info plasmid could not be recombined in the absence of λRed. In donor DNA-free groups, there was no donor DNA fragment to recombine the info plasmid. In all the four cases, information could not be rewritten successfully as suggested by the sequenced DNA sequence. When information was successfully rewritten, green fluorescent color was clearly observed in the rewritten strains, suggesting that the expression of GFP is a good indicator for successful information rewriting. The binary codes of text message “IT WAS THE WORST OF TIMES” are encoded into the DNA sequence using the Base64 coding algorithm. After information rewriting, the DNA sequence carries the rewritten message “IT WAS THE BEST OF TIMES”.

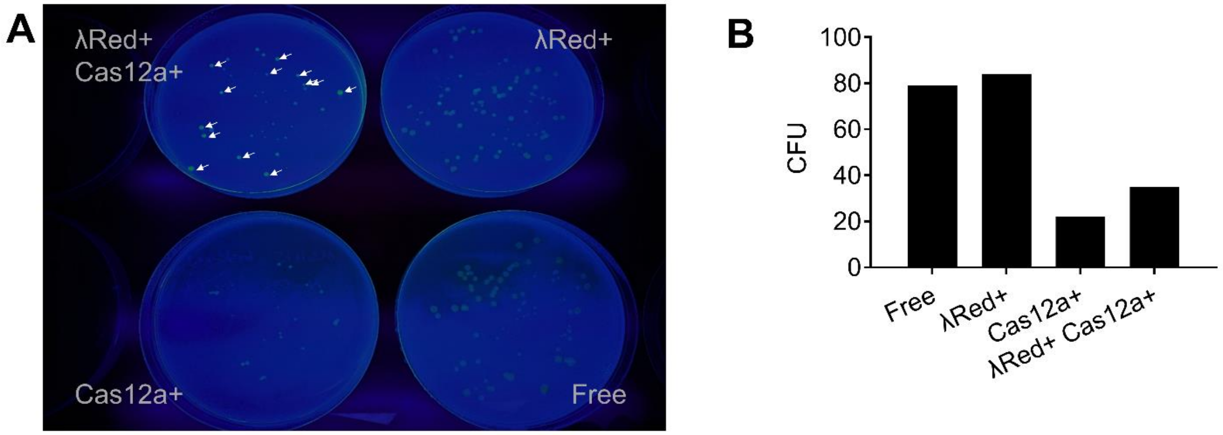

Fig. S5. Performances of Cas12a and λRed for information rewriting encoded by 15-ary Huffman algorithm. (A) Optical images of Cas12a- and λRed-modified, λRed-modified, Cas12a-modified and control strains under UV light, respectively. **(B)** Colony-forming units (CFU) of strains under different treatments. The statistical data suggests that Cas12a is necessary for cutting the info plasmid and λRed is helpful for recombining donor DNA with the info plasmid.

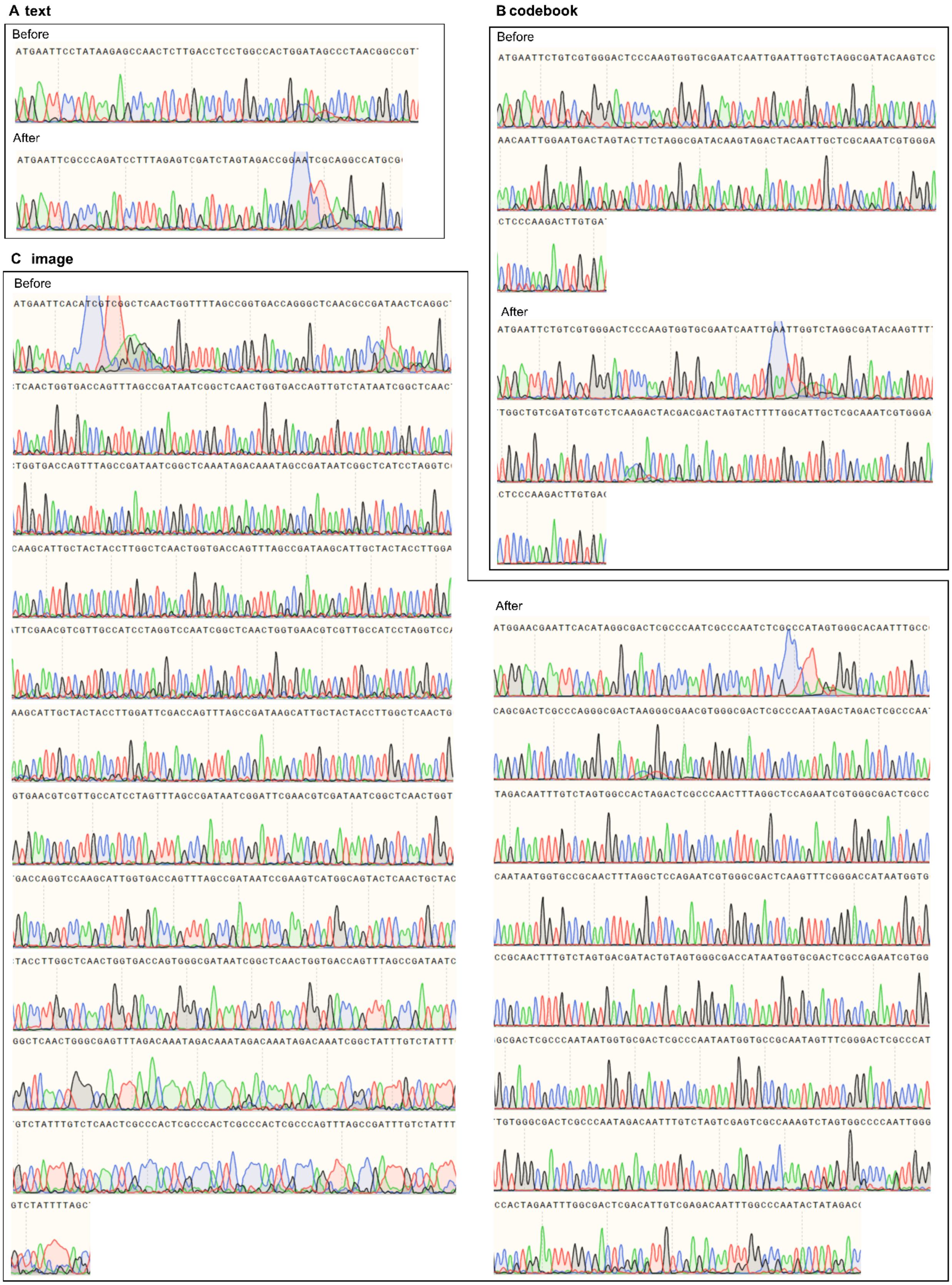
A text
Before
After
B codebook
Before
After
C image
Before
After

Fig. S6. First generation sequencing of DNA sequences encoded by 15-ary Huffman algorithm before and after information rewriting. DNA sequences of **(A)** text, **(B)** codebook and **(C)** image information before and after information rewriting. DNA sequences are sequenced by the Sanger sequencer using the primer M13F. For the codebook, before row switch, the ASCII codes were [42, 32, 42, 32, 42, 32, 42, 32, 42, 13, 10, 42, 32, 49, 32, 50, 32, 51, 32, 42, 13, 10, 42, 32, 206, 177, 32, 206, 178, 32, 206, 179, 32, 42, 13, 10, 42, 32, 97, 32, 98, 32, 99, 32, 42, 13, 10, 42, 32, 42, 32, 42, 32, 42, 32, 42, 13, 10] and the DNA sequence was 0,TGTCGTGGGACTCCCAAGTGGTGCGAATCAATTGAATTGGTCTAGGCGATACAAGTCCAACAATTGGAATGACTAGTACTTCTAGGCGATACAAGTAGACTACAATTGCTCGCAAATCGTGGGACTCCCAAGACTTGTGA,140.0. After row switch, the revised ASCII codes were [42, 32, 42, 32, 42, 32, 42, 32, 42, 13, 10, 42, 32, 49, 32, 50, 32, 51, 32, 42, 13, 10, 42, 32, 97, 32, 98, 32, 99, 32, 42, 13, 10, 42, 32, 206, 177, 32, 206, 178, 32, 206, 179, 32, 42, 13, 10, 42, 32, 42, 32, 42, 32, 42, 32, 42, 13, 10] and the revised DNA sequence was 0,TGTCGTGGGACTCCCAAGTGGTGCGAATCAATTGAATTGGTCTAGGCGATACAAGTTTTGGCTGTCGATGTCGTCTCAAGACTACGACGACTAGTACTTTTGGCATTGCTCGCAAATCGTGGGACTCCCAAGACTTGTGA,140.0. Therefore, the row switch operation was achieved by replacing the sequence “CCAACAATTGGAATGACTAGTACTTCTAGGCGATACAAGTAGACTACA” in the original DNA sequence with “TTTGGCTGTCGATGTCGTCTCAAGACTACGACGACTAGTACTTTTGGC”.

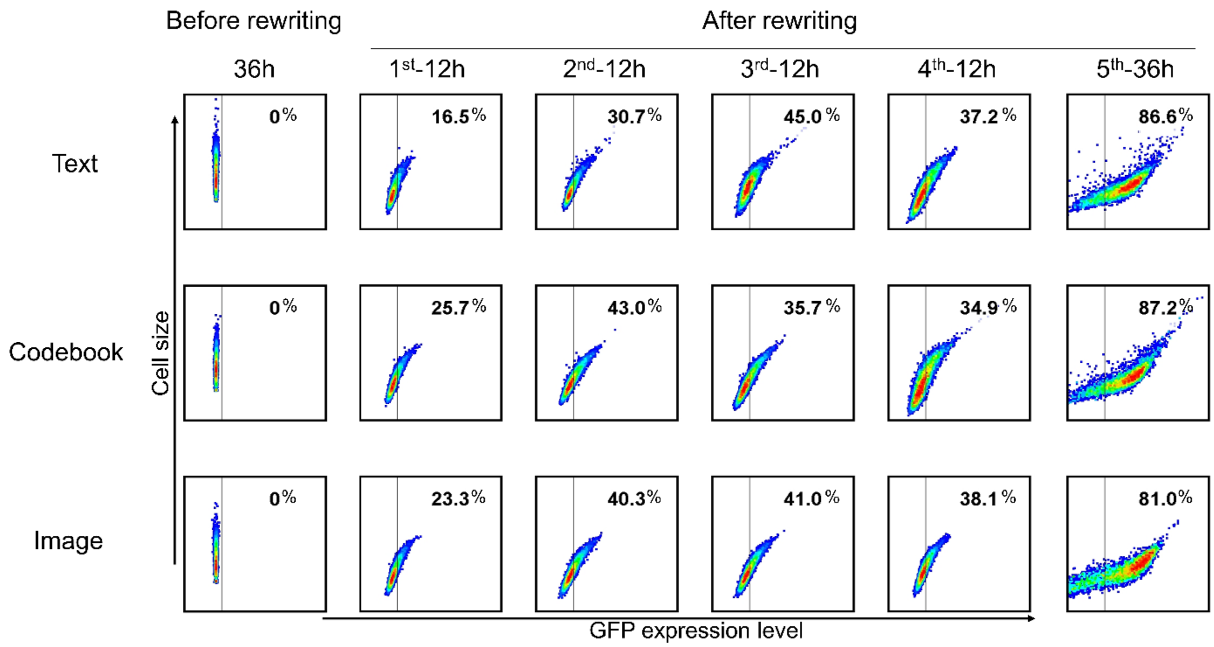

Fig. S7. Flow cytometry analysis of cells before and after information rewriting. After information rewriting, the bacteria were inoculated every 12 hours for four times, during which bacteria grow and proliferate under an optimal culture condition at 30°C. Although the level of GFP expression was low at the beginning, green fluorescence could be detected in the rewritten strains and GFP expression becomes higher from generation to generation.

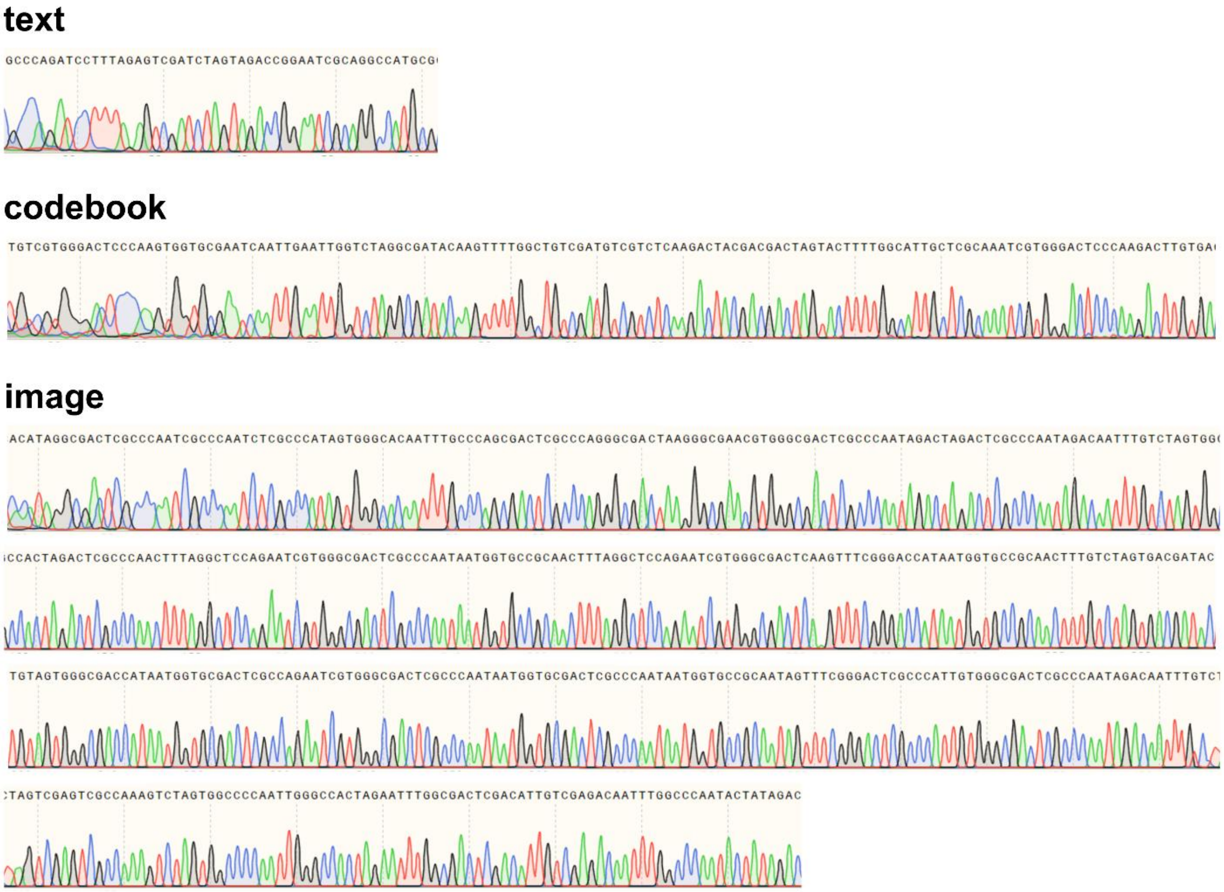

Fig S8. First generation sequencing of DNA sequences retrieved from fluorescent stains after 252 generations of growth. Specifically, a rewritten fluorescent strain with edited information is re-inoculated every 12 h for four times, during which bacteria are allowed to proliferate under optimal culture conditions. After the fifth inoculation, the bacteria still show high GFP expression level and the purified plasmid from the culture solution is sequenced with a single signal of Sanger sequencing. The sequencing results demonstrated that the rewritten information decoded from the DNA fragment from fluorescent strains after 252 generations of growth is 100% correct, indicating that the DNA-based information stored in bacteria is robust and could be passed on to future generations.

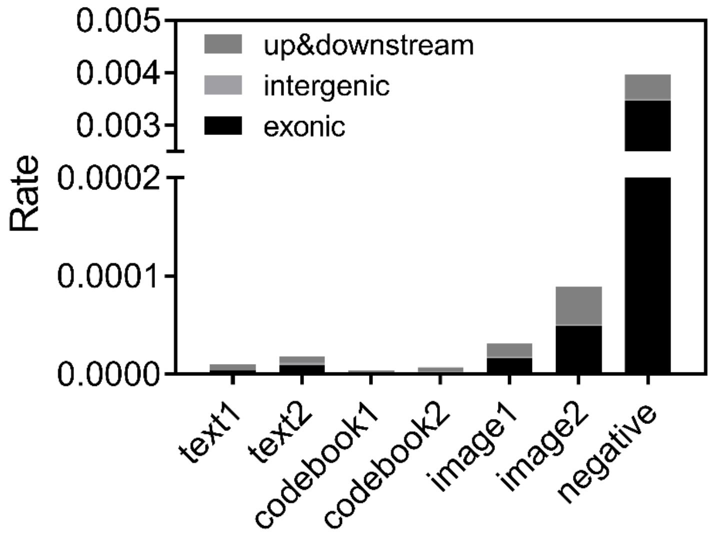

Fig. S9. The rate distributions of single nucleotide variants among the total genome of information-encoded *E.coli* strains genome. The *E. coli* strains MG1655HT (text1), MG1655HNT (text2), MG1655HC (codebook1), MG1655HNC (codebook2), MG1655HI (image1), and MG1655HNI (image2) which were encoded with different information before and after rewriting, were stored at 4°C with 20% glycerin for 16 months. The negative control strain (negative) was MG1655 strain only treated by CRISPR-Cas12a without guided RNA and donor DNA. All strains were inoculated in 5 mL LB media, proliferated at 30°C for 20 hours, collected by centrifugation for next-generation sequencing. By comparing the sequencing results with the reference sequence of MG1655 complete genome (NCBI Reference Sequence: NC_000913.3, https://www.ncbi.nlm.nih.gov/nuccore/556503834?report=fasta), the rate of single nucleotide variants among the complete genome were calculated and classified as upstream and downstream (up&downstream), intergenic and exonic, by functional area location of mutant base pair. The average rate of single nucleotide variants among the rewritten information-encoded strains was 0.000030, which was 0.76% of the negative control group, indicating that there was lower genome-wide off-target effect in the information rewriting process.

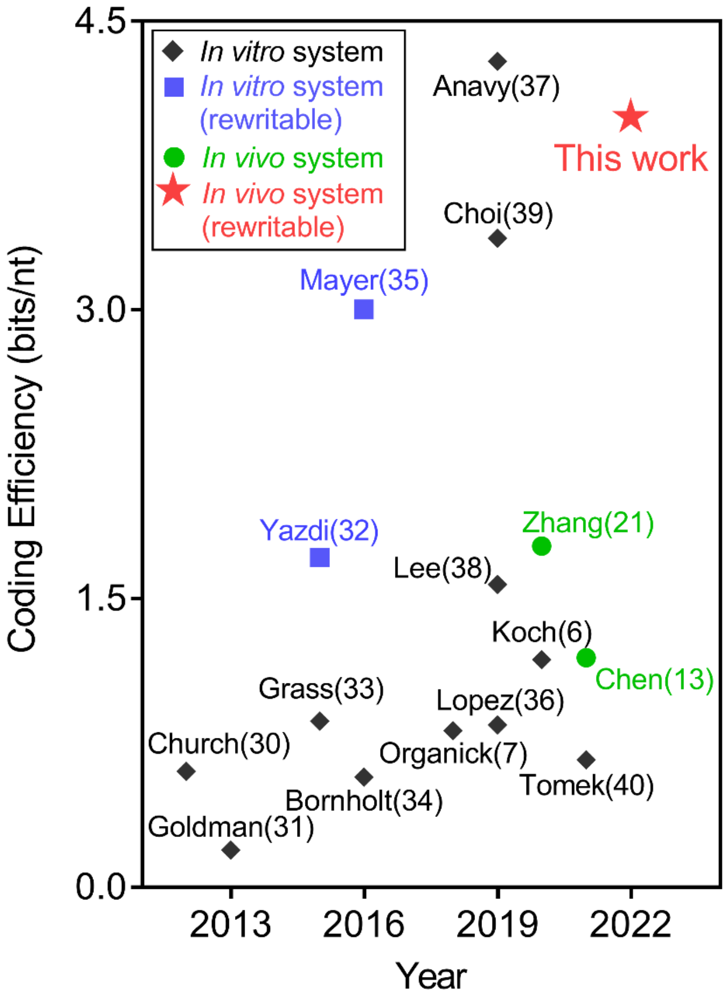

Fig. S10. Comparison of the storage density of our *in vivo* rewriting system with other DNA-based data storage approaches. The storage density of information storing and rewriting in the dual-plasmid system outperforms other DNA-based information technology researches.

Table S1. DNA sequences in the plasmids and donor DNA sequences. DNA sequences, which carry the digital information, are capitalized in bold. PAM site (TTTN) and subsequent nucleotides presented in lowercase bold letters are recognized by crRNA.

Name	Nucleotide Sequence (5’ to 3’)	Comment
p46Cpf1-OP2 （*ref.24*）	GTCGAGCATGCGACGTCTTAAGACCCACTTTCACATTTAAGTTGTTTTTCTAATCCGCATATGATCAATTCAAG<br>GCCGAATAAGAAGGCTGGCTCTGCACCTTGGTGATCAAATAATTCGATAGCTTGTCGTAATAATGGCGGCATAC<br>TATCAGTAGTAGGTGTTTCCCTTTCTTCTTTAGCGACTTGATGCTCTTGATCTTCCAATACGCAACCTAAAGTAA<br>AATGCCCCACAGCGCTGAGTGCATATAATGCATTCTCTAGTGAAAAACCTTGTTGGCATAAAAAGGCTAATTG<br>ATTTTCGAGAGTTTCATACTGTTTTTCTGTAGGCCGTGTACCTAAATGTACTTTTGCTCCATCGCGATGACTTAG<br>TAAAGCACATCTAAAACTTTTAGCGTTATTACGTAAAAAATCTTGCCAGCTTTCCCCTTCTAAAGGGCAAAAGT<br>GAGTATGGTGCCTATCTAACATCTCAATGGCTAAGGCGTCGAGCAAAGCCCGCTTATTTTTTACATGCCAATAC<br>AATGTAGGCTGCTCTACACCTAGCTTCTGGGCGAGTTTACGGGTTGTTAAACCTTCGATTCCGACCTCATTAAG<br>CAGCTCTAATGCGCTGTTAATCACTTTACTTTTATCTAATCTAGACATCATTAATTCCTAATTTTTGTTGACACTC<br>TATCGTTGATAGAGTTATTTTACCACTCCCTATCAGTGATAGAGAAAAGAATTCAAAAGATCTAAAGAGGAGA<br>AAGGCTCGAGAAAAAAATGGATATTAATACTGAAACTGAGATCAAGCAAAAGCATTCACTAACCCCCTTTCCTG<br>TTTTCCTAATCAGCCCGGCATTTCGCGGGCGATATTTTCACAGCTATTTCAGGAGTTCAGCCATGAACGCTTATT<br>ACATTCAGGATCGTCTTGAGGCTCAGAGCTGGGCGCGTCACTACCAGCAGCTCGCCCGTGAAGAGAAAGAG<br>GCAGAACTGGCAGACGACATGGAAAAAGGCCTGCCCCAGCACCTGTTTGAATCGCTATGCATCGATCATTTGC<br>AACGCCACGGGGCCAGCAAAAAATCCATTACCCGTGCGTTTGATGACGATGTTGAGTTTCAGGAGCGCATGG<br>CAGAACACATCCGGTACATGGTTGAAACCATTGCTCACCACCAGGTTGATATTGATTCAGAGGTATAAAACGA<br>ATGAGTACTGCACTCGCAACGCTGGCTGGGAAGCTGGCTGAACGTGTCGGCATGGATTCTGTCGACCCACAG<br>GAACTGATCACCACTCTTCGCCAGACGGCATTTAAAGGTGATGCCAGCGATGCGCAGTTCATCGCATTACTGA<br>TCGTTGCCAACCAGTACGGCCTTAATCCGTGGACGAAAGAAATTTACGCCTTTCCTGATAAGCAGAATGGCAT<br>CGTTCCGGTGGTGGGCGTTGATGGCTGGTCCCGCATCATCAATGAAAACCAGCAGTTTGATGGCATGGACTTT<br>GAGCAGGACAATGAATCCTGTACATGCCGGATTTACCGCAAGGACCGTAATCATCCGATCTGCGTTACCGAAT<br>GGATGGATGAATGCCGCCGCGAACCATTCAAAACTCGCGAAGGCAGAGAAATCACGGGGCCGTGGCAGTCG<br>CATCCCAAACGGATGTTACGTCATAAAGCCATGATTCAGTGTGCCCGTCTGGCCTTCGGATTTGCTGGTATCTA<br>TGACAAGGATGAAGCCGAGCGCATTGTCGAAAATACTGCATACACTGCAGAACGTCAGCCGGAACGCGACAT<br>CACTCCGGTTAACGATGAAACCATGCAGGAGATTAACACTCTGCTGATCGCCCTGGATAAAACATGGGATGAC<br>GACTTATTGCCGCTCTGTTCCCAGATATTTCGCCGCGACATTCGTGCATCGTCAGAACTGACACAGGCCGAAG<br>CAGTAAAAGCTCTTGGATTCCTGAAACAGAAAGCCGCAGAGCAGAAGGTGGCAGCATGACACCGGACATTAT<br>CCTGCAGCGTACCGGGATCGATGTGAGAGCTGTCGAACAGGGGGATGATGCGTGGCACAAATTACGGCTCGG<br>CGTCATCACCGCTTCAGAAGTTCACAACGTGATAGCAAAACCCCGCTCCGGAAAGAAGTGGCCTGACATGAA<br>AATGTCCTACTTCCACACCCTGCTTGCTGAGGTTTGCACCGGTGTGGCTCCGGAAGTTAACGCTAAAGCACTG<br>GCCTGGGGAAAACAGTACGAGAACGACGCCAGAACCCTGTTTGAATTCACTTCCGGCGTGAATGTTACTGAA<br>TCCCCGATCATCTATCGCGACGAAAGTATGCGTACCGCCTGCTCTCCCGATGGTTTATGCAGTGACGGCAACG<br>GCCTTGAACTGAAATGCCCGTTTACCTCCCGGGATTTCATGAAGTTCCGGCTCGGTGGTTTCGAGGCCATAAA	Help plasmid (Addgene plasmid # 98592)

Name	Nucleotide Sequence (5’ to 3’)	Comment
	GTCAGCTTACATGGCCCAGGTGCAGTACAGCATGTGGGTGACGCGAAAAAATGCCTGGTACTTTGCCAACTAT<br>GACCCGCGTATGAAGCGTGAAGGCCTGCATTATGTCGTGATTGAGCGGGATGAAAAGTACATGGCGAGTTTTG<br>ACGAGATCGTGCCGGAGTTCATCGAAAAAATGGACGAGGCACTGGCTGAAATTGGTTTTGTATTTGGGGAGC<br>AATGGCGATGACGCATCCTCACGATAATATCCGGGTAGGCGCAATCACTTTCGTCTACTCCGTTACAAAGCGAG<br>GCTGGGTATTTCCCGGCCTTTCTGTTATCCGAAATCCACTGAAAGCACAGCGGCTGGCTGAGGAGATAAATAA<br>TAAACGAGGGGCTGTATGCACAAAGCATCTTCTGTTGAGTTAAGAACGAGTATCGAGATGGCACATAGCCTTG<br>CTCAAATTGGAATCAGGTTTGTGCCAATACCAGTAGAAACAGACGAAGAATCCATGGAGCTCATATGGTACCG<br>GTATGGACAGTTTTCCCTTTGATATGTAACGGTGAACAGTTGTTCTACTTTTGTTTGTTAGTCTTGATGCTTCAC<br>TGATAGATACAAGAGCCATAAGAACCTCAGATCCTTCCGTATTTAGCCAGTATGTTCTCTAGTGTGGTTCGTTG<br>TTTTTGCGTGAGCCATGAGAACGAACCATTGAGATCATACTTACTTTGCATGTCACTCAAAAATTTTGCCTCAA<br>AACTGGTGAGCTGAATTTTTGCAGTTAAAGCATCGTGTAGTGTTTTTCTTAGTCCGTTATGTAGGTAGGAATCT<br>GATGTAATGGTTGTTGGTATTTTGTCACCATTCATTTTTATCTGGTTGTTCTCAAGTTCGGTTACGAGATCCATTT<br>GTCTATCTAGTTCAACTTGGAAAATCAACGTATCAGTCGGGCGGCCTCGCTTATCAACCACCAATTTCATATTG<br>CTGTAAGTGTTTAAATCTTTACTTATTGGTTTCAAAACCCATTGGTTAAGCCTTTTAAACTCATGGTAGTTATTT<br>TCAAGCATTAACATGAACTTAAATTCATCAAGGCTAATCTCTATATTTGCCTTGTGAGTTTTCTTTTGTGTTAGT<br>TCTTTTAATAACCACTCATAAATCCTCATAGAGTATTTGTTTTCAAAAGACTTAACATGTTCCAGATTATATTTTA<br>TGAATTTTTTTAACTGGAAAAGATAAGGCAATATCTCTTCACTAAAAACTAATTCTAATTTTTCGCTTGAGAAC<br>TTGGCATAGTTTGTCCACTGGAAAATCTCAAAGCCTTTAACCAAAGGATTCCTGATTTCCACAGTTCTCGTCAT<br>CAGCTCTCTGGTTGCTTTAGCTAATACACCATAAGCATTTTCCCTACTGATGTTCATCATCTGAACGTATTGGTT<br>ATAAGTGAACGATACCGTCCGTTCTTTCCTTGTAGGGTTTTCAATCGTGGGGTTGAGTAGTGCCACACAGCATA<br>AAATTAGCTTGGTTTCATGCTCCGTTAAGTCATAGCGACTAATCGCTAGTTCATTTGCTTTGAAAACAACTAATT<br>CAGACATACATCTCAATTGGTCTAGGTGATTTTAATCACTATACCAATTGAGATGGGCTAGTCAATGATAATTAC<br>TAGTCCTTTTCCTTTGAGTTGTGGGTATCTGTAAATTCTGCTAGACCTTTGCTGGAAAACTTGTAAATTCTGCTA<br>GACCCTCTGTAAATTCCGCTAGACCTTTGTGTGTTTTTTTTGTTTATATTCAAGTGGTTATAATTTATAGAATAAA<br>GAAAGAATAAAAAAAGATAAAAAGAATAGATCCCAGCCCTGTGTATAACTCACTACTTTAGTCAGTTCCGCAG<br>TATTACAAAAGGATGTCGCAAACGCTGTTTGCTCCTCTACAAAACAGACCTTAAAACCCTAAAGGCTTAAGTA<br>GCACCCTCGCAAGCTCGGTTGCGGCCGCAATCGGGCAAATCGCTGAATATTCCTTTTGTCTCCGACCATCAGG<br>CACCTGAGTCGCTGTCTTTTTCGTGACATTCAGTTCGCTGCGCTCACGGCTCTGGCAGTGAATGGGGGTAAAT<br>GGCACTACAGGCGCCTTTTATGGATTCATGCAAGGAAACTACCCATAATACAAGAAAAGCCCGTCACGGGCTT<br>CTCAGGGCGTTTTATGGCGGGTCTGCTATGTGGTGCTATCTGACTTTTTGCTGTTCAGCAGTTCCTGCCCTCTG<br>ATTTTCCAGTCTGACCACTTCGGATTATCCCGTGACAGGTCATTCAGACTGGCTAATGCACCCAGTAAGGCAG<br>CGGTATCATCAACGGGGTCTGACGCTCAGTGGAACGAAAACTCACGTTAAGGGCCTAGGGACTCCTGTTGAT<br>AGATCCAGTAATGACCTCAGAACTCCATCTGGATTTGTTCAGAACGCTCGGTTGCCGCCGGGCGTTTTTTATTG<br>GTGAGAATCATCGATTTATTATGACAACTTGACGGCTACATCATTCACTTTTTCTTCACAACCGGCACGGAACT<br>CGCTCGGGCTGGCCCCGGTGCATTTTTTAAATACCCGCGAGAAGTAGAGTTGATCGTCAAAACCAACATTGCG<br>ACCGACGGTGGCGATAGGCATCCGGGTGGTGCTCAAAAGCAGCTTCGCCTGGCTGATACGTTGGTCCTCGCG	

Name	Nucleotide Sequence (5’ to 3’)	Comment
	CCAGCTTAAGACGCTAATCCCTAACTGCTGGCGGAAAAGATGTGACAGACGCGACGGCGACAAGCAAACATG<br>CTGTGCGACGCTGGCGATATCAAAATTGCTGTCTGCCAGGTGATCGCTGATGTACTGACAAGCCTCGCGTACC<br>CGATTATCCATCGGTGGATGGAGCGACTCGTTAATCGCTTCCATGCGCCGCAGTAACAATTGCTCAAGCAGATT<br>TATCGCCAGCAGCTCCGAATAGCGCCCTTCCCCTTGCCCGGCGTTAATGATTTGCCCAAACAGGTCGCTGAAA<br>TGCGGCTGGTGCGCTTCATCCGGGCGAAAGAACCCCGTATTGGCAAATATTGACGGCCAGTTAAGCCATTCAT<br>GCCAGTAGGCGCGCGGACGAAAGTAAACCCACTGGTGATACCATTCGCGAGCCTCCGGATGACGACCGTAGT<br>GATGAATCTCTCCTGGCGGGAACAGCAAAATATCACCCGGTCGGCAAACAAATTCTCGTCCCTGATTTTTCAC<br>CACCCCCTGACCGCGAATGGTGAGATTGAGAATATAACCTTTCATTCCCAGCGGTCGGTCGATAAAAAAATCG<br>AGATAACCGTTGGCCTCAATCGGCGTTAAACCCGCCACCAGATGGGCATTAAACGAGTATCCCGGCAGCAGG<br>GGATCATTTTGCGCTTCAGCCATACTTTTCATACTCCCGCCATTCAGAGAAGAAACCAATTGTCCATATTGCATC<br>AGACATTGCCGTCACTGCGTCTTTTACTGGCTCTTCTCGCTAACCAAACCGGTAACCCCGCTTATTAAAAGCAT<br>TCTGTAACAAAGCGGGACCAAAGCCATGACAAAAACGCGTAACAAAAGTGTCTATAATCACGGCAGAAAAG<br>TCCACATTGATTATTTGCACGGCGTCACACTTTGCTATGCCATAGCATTTTTATCCATAAGATTAGCGGATCCTA<br>CCTGACGCTTTTTATCGCAACTCTCTACTGTTTCTCCATGTGCACGAATTCAAAAGATCTAAAGAGGAGAAAG<br>GATCTATGTCTATCTACCAGGAATTCGTTAACAAATACTCTCTGTCTAAAACCCTGCGTTTCGAACTGATCCCG<br>CAGGGTAAAACCCTGGAAAACATCAAAGCTCGTGGTCTGATCCTGGACGACGAAAAACGTGCTAAAGACTAC<br>AAAAAAGCTAAACAGATCATCGACAAATACCACCAGTTCTTCATCGAAGAAATCCTCTCGTCTGTCTGCATCA<br>GCGAAGACCTGCTGCAGAACTACTCGGACGTGTACTTCAAACTAAAAAAATCTGACGACGACAACCTGCAGA<br>AAGACTTCAAATCTGCTAAAGACACCATCAAAAAACAGATCTCTGAATACATCAAAGACTCTGAAAAATTCA<br>AAAACCTGTTCAACCAGAACCTGATCGACGCTAAAAAAGGTCAGGAATCTGACCTGATCCTGTGGCTGAAAC<br>AGTCTAAAGACAACGGTATCGAACTGTTCAAAGCTAACTCTGACATCACCGACATCGACGAAGCTCTGGAAA<br>TCATCAAATCTTTCAAAGGTTGGACCACCTACTTCAAAGGTTTCCACGAAAACCGTAAAAACGTTTACTCTTC<br>TAACGACATCCCGACCTCTATCATCTACCGTATCGTTGACGACAACCTGCCGAAATTCCTGGAAAACAAAGCT<br>AAATACGAATCTCTGAAAGACAAAGCTCCGGAAGCTATCAACTACGAACAGATCAAAAAAGACCTGGCTGAA<br>GAACTGACCTTCGACATCGACTACAAAACCTCTGAAGTTAACCAGCGTGTTTTCTCTCTGGACGAAGTTTTCG<br>AAATCGCTAACTTCAACAACTACCTGAACCAGTCTGGTATCACCAAATTCAACACCATCATCGGTGGTAAATT<br>CGTTAACGGTGAAAACACCAAACGTAAAGGTATCAACGAATACATCAACCTGTACTCTCAGCAGATCAACGA<br>CAAAACCCTGAAAAAATACAAAATGTCTGTTCTGTTCAAACAGATCCTGTCTGACACCGAATCTAAATCTTTC<br>GTTATCGACAAACTGGAAGACGACTCTGACGTTGTTACCACCATGCAGTCTTTCTACGAACAGATCGCTGCTT<br>TCAAAACCGTTGAAGAAAAATCTATCAAAGAAACCCTGTCTCTGCTGTTCGACGACCTGAAAGCTCAGAAAC<br>TGGACCTGTCTAAAATCTACTTCAAAAACGACAAATCTCTGACCGACCTCTCTCAGCAGGTATTCGACGACTA<br>CAGCGTTATCGGTACCGCTGTTCTGGAATACATCACCCAGCAGATCGCTCCGAAAAACCTGGACAACCCGTCT<br>AAAAAAGAACAGGAACTGATCGCTAAAAAAACCGAAAAAGCTAAATACCTGTCTCTGGAAACCATCAAACT<br>GGCTCTGGAAGAATTCAACAAACACCGTGACATCGACAAACAGTGCCGTTTCGAAGAAATCCTGGCTAACTT<br>CGCTGCTATCCCGATGATCTTCGACGAAATCGCTCAGAACAAAGACAACCTGGCTCAGATCTCTATCAAATAC<br>CAGAACCAGGGTAAAAAAGACCTGCTGCAGGCTTCTGCTGAAGACGACGTTAAAGCTATCAAAGACCTGCTG	

Name	Nucleotide Sequence (5’ to 3’)	Comment
	GACCAGACCAACAACCTGCTGCACAAACTGAAAATCTTCCACATCTCTCAGTCTGAAGACAAAGCTAACATC<br>CTGGACAAAGACGAACACTTCTACCTGGTTTTCGAAGAATGCTACTTCGAACTGGCTAACATCGTTCCGCTGT<br>ACAACAAAATCCGTAACTACATCACCCAGAAACCGTACTCTGACGAAAAATTCAAACTGAACTTCGAAAACT<br>CTACCCTGGCTAACGGTTGGGACAAAAACAAAGAACCGGACAACACCGCTATCCTGTTCATCAAAGACGACA<br>AATACTACCTGGGTGTTATGAACAAAAAAAACAACAAAATCTTCGACGACAAAGCTATCAAAGAAAACAAAG<br>GTGAAGGTTACAAAAAAATCGTTTACAAACTGCTGCCGGGTGCTAACAAAATGCTGCCGAAAGTTTTCTTCTC<br>TGCTAAATCTATCAAATTCTACAACCCGTCTGAAGACATCCTGCGTATCCGTAACCACTCTACCCACACCAAAA<br>ACGGTTCTCCGCAGAAAGGTTACGAAAAATTCGAATTCAACATCGAAGACTGCCGTAAATTCATCGACTTCTA<br>CAAACAGTCTATCTCTAAACACCCGGAATGGAAAGACTTCGGTTTCCGTTTCTCTGACACCCAGCGTTACAAC<br>TCTATCGACGAATTCTACCGTGAAGTTGAAAACCAGGGTTACAAACTGACCTTCGAAAACATCTCTGAATCTT<br>ACATCGACTCTGTTGTTAACCAGGGTAAACTGTACCTGTTCCAGATCTACAACAAAGACTTCTCTGCTTACTCT<br>AAAGGTCGTCCGAACCTGCACACCCTGTACTGGAAAGCTCTGTTCGACGAACGTAACCTGCAGGACGTTGTT<br>TACAAACTGAACGGTGAAGCTGAACTGTTCTACCGTAAACAGTCTATCCCGAAAAAAATCACCCACCCGGCT<br>AAAGAAGCTATCGCTAACAAAAACAAAGACAACCCGAAAAAAGAATCTGTTTTCGAATACGACCTGATCAAA<br>GACAAACGTTTCACCGAAGACAAATTCTTCTTCCACTGCCCGATCACCATCAACTTCAAATCTTCTGGTGCTA<br>ACAAATTCAACGACGAAATCAACCTGCTGCTGAAAGAAAAAGCTAACGACGTTCACATCCTGTCTATCGACC<br>GTGGTGAACGTCACCTGGCTTACTACACCCTGGTTGACGGTAAAGGTAACATCATCAAACAGGACACCTTCA<br>ACATCATCGGTAACGACCGTATGAAAACCAACTACCACGACAAACTGGCTGCTATCGAAAAAGACCGTGACT<br>CTGCTCGTAAAGACTGGAAAAAAATCAACAACATCAAAGAAATGAAAGAAGGTTACCTGTCTCAGGTTGTTC<br>ACGAAATCGCTAAACTGGTTATAGAATACAATGCGATAGTTGTATTTGAAGACCTGAACTTCGGCTTCAAACGT<br>GGTCGTTTCAAAGTTGAAAAACAGGTTTACCAGAAACTGGAAAAAATGCTGATCGAAAAACTGAACTACCTG<br>GTTTTCAAAGACAACGAATTCGACAAAACCGGTGGTGTTCTGCGTGCTTACCAGCTGACCGCTCCGTTCGAA<br>ACCTTCAAAAAAATGGGTAAACAGACCGGTATCATCTACTACGTTCCGGCTGGTTTCACCTCTAAAATCTGCC<br>CTGTTACTGGCTTCGTTAACCAGCTCTACCCGAAATACGAATCTGTTTCTAAATCTCAGGAATTCTTCTCTAAAT<br>TCGACAAAATCTGCTACAACCTGGACAAAGGTTACTTCGAATTCTCTTTCGACTACAAAAACTTCGGTGACAA<br>AGCTGCTAAAGGTAAATGGACCATCGCTTCTTTCGGTTCTCGTCTGATCAACTTCCGTAACTCTGACAAAAAC<br>CACAACTGGGACACCCGTGAAGTTTACCCGACCAAAGAACTGGAAAAACTGCTGAAAGACTACTCTATCGAA<br>TACGGTCACGGTGAATGCATCAAAGCTGCTATCTGCGGTGAATCTGACAAAAAATTCTTCGCTAAACTGACCT<br>CTGTTCTGAACACCATCCTGCAGATGCGTAACTCTAAAACCGGTACCGAACTGGACTACCTGATCTCTCCGGT<br>TGCTGACGTTAACGGTAACTTCTTCGACTCTCGTCAGGCTCCGAAAAACATGCCGCAGGACGCTGACGCTAA<br>CGGTGCTTACCACATCGGTCTGAAAGGTCTGATGCTGCTGGGTCGTATCAAAAACAACCAGGAAGGTAAAAA<br>ACTGAACCTGGTTATCAAAAACGAAGAATACTTCGAATTCGTTCAGAACCGTAACAACTAATAACCAGGCATC<br>AAATAAAACGAAAGGCTCAGTCGAAAGACTGGGCCTTTCGTTTTATCTGTTGTTTGTCGGTGAACGCTCTCTA<br>CTAGAGTCACACTGGCTCACCTTCGGGTGGGCCTTTCTGCGGTGCACAAGCGAGCTCGATATCAAATTACGCC<br>CCGCCCTGCCACTCATCGCAGTACTGTTGTAATTCATTAAGCATTCTGCCGACATGGAAGCCATCACAAACGG<br>CATGATGAACCTGAATCGCCAGCGGCATCAGCACCTTGTCGCCTTGCGTATAATATTTGCCCATGGTGAAAACG	

Name	Nucleotide Sequence (5’ to 3’)	Comment
	GGGGCGAAGAAGTTGTCCATATTGGCCACGTTTAAATCAAAACTGGTGAAACTCACCCAGGGATTGGCTGAG<br>ACGAAAAACATATTCTCAATAAACCCTTTAGGGAAATAGGCCAGGTTTTCACCGTAACACGCCACATCTTGCG<br>AATATATGTGTAGAAACTGCCGGAAATCGTCGTGGTATTCACTCCAGAGCGATGAAAACGTTTCAGTTTGCTC<br>ATGGAAAACGGTGTAACAAGGGTGAACACTATCCCATATCACCAGCTCACCGTCTTTCATTGCCATACGAAAT<br>TCCGGATGAGCATTCATCAGGCGGGCAAGAATGTGAATAAAGGCCGGATAAAACTTGTGCTTATTTTTCTTTAC<br>GGTCTTTAAAAAGGCCGTAATATCCAGCTGAACGGTCTGGTTATAGGTACATTGAGCAACTGACTGAAATGCC<br>TCAAAATGTTCTTTACGATGCCATTGGGATATATCAACGGTGGTATATCCAGTGATTTTTTTCTCCATTTTAGCTT<br>CCTTAGCTCCTGAAAATCTCGATAACTCAAAAAATACGCCCGGTAGTGATCTTATTTCATTATGGTGAAAGTTG<br>GAACCTCTTACGTGCCGATCA	
pUC57-P*tac*GFP	TCGCGCGTTTCGGTGATGACGGTGAAAACCTCTGACACATGCAGCTCCCGGAGACTGTCACAGCTTGTCTGT<br>AAGCGGATGCCGGGAGCAGACAAGCCCGTCAGGGCGCGTCAGCGGGTGTTGGCGGGTGTCGGGGCTGGCTT<br>AACTATGCGGCATCAGAGCAGATTGTACTGAGAGTGCACCATATGCGGTGTGAAATACCGCACAGATGCGTAA<br>GGAGAAAATACCGCATCAGGCGCCATTCGCCATTCAGGCTGCGCAACTGTTGGGAAGGGCGATCGGTGCGGG<br>CCTCTTCGCTATTACGCCAGCTGGCGAAAGGGGGATGTGCTGCAAGGCGATTAAGTTGGGTAACGCCAGGGT<br>TTTCCCAGTCACGACGTTGTAAAACGACGGCCAGTGAATTGACGCGTATTGGGATGAATTCCGATCCCGCGAA<br>ATTAATACGAGCTGTTGACAATTAATCATCGGCTCGTATAATGTGTGGAATTGTGAGCGGATAACAATTCCCCTC<br>TAGAAATAATTTTGTTTAACTTTAAGAAGGAGATATACATATGGCTAGCAAAGGAGAAGAACTTTTCACTGGA<br>GTTGTCCCAATTCTTGTTGAATTAGATGGTGATGTTAATGGTCACAAATTTTCTGTCAGTGGAGAGGGTGAAG<br>GTGATGCAACATACGGAAAACTTACCCTTAAATTTATTTGCACTACTGGAAAACTACCTGTTCCATGGCCAACA<br>CTTGTCACTACTTTAACTTATGGTGTTCAATGCTTTTCCCGTTATCCGGATCATATGAAACGTCATGACTTTTTC<br>AAGAGTGCCATGCCCGAAGGTTATGTACAGGAACGCACTATATCTTTCAAAGATGACGGTAACTACAAGACGC<br>GTGCTGAAGTCAAGTTTGAAGGTGATACCCTTGTTAATCGTATCGAGTTAAAAGGTATTGATTTTAAAGAAGAT<br>GGAAACATTCTCGGACACAAACTCGAGTACAACTATAACTCACACAATGTATACATCACGGCAGACAAACAA<br>AAGAATGGAATCAAGGCTAACTTCAAAATTCGCCACAACATTGAAGATGGATCCGTTCAACTAGCAGACCATT<br>ATCAACAAAATACTCCAATTGGCGATGGCCCTGTCCTTTTACCAGACAACCATTACCTGTCGACACAATCTGCC<br>CTTTCGAAAGATCCCAACGAAAAGCGTGACCACATGGTCCTTCTTGAGTTTGTAACTGCTGCTGGTATTACAC<br>ATGGCATGGATTAGAAGCTTATCCCAATGGCGCGCCGAGCTTGGCGTAATCATGGTCATAGCTGTTTCCTGTGT<br>GAAATTGTTATCCGCTCACAATTCCACACAACATACGAGCCGGAAGCATAAAGTGTAAAGCCTGGGGTGCCTA<br>ATGAGTGAGCTAACTCACATTAATTGCGTTGCGCTCACTGCCCGCTTTCCAGTCGGGAAACCTGTCGTGCCAG<br>CTGCATTAATGAATCGGCCAACGCGCGGGGAGAGGCGGTTTGCGTATTGGGCGCTGTTCCGCTTCCTCGCTCA<br>CTGACTCGCTGCGCTCGGTCGTTCGGCTGCGGCGAGCGGTATCAGCTCACTCAAAGGCGGTAATACGGTTATC<br>CACAGAATCAGGGGATAACGCAGGAAAGAACATGTGAGCAAAAGGCCAGCAAAAGGCCAGGAACCGTAAA<br>AAGGCCGCGTTGCTGGCGTTTTTCCATAGGCTCCGCCCCCCTGACGAGCATCACAAAAATCGACGCTCAAGTC<br>AGAGGTGGCGAAACCCGACAGGACTATAAAGATACCAGGCGTTTCCCCCTGGAAGCTCCCTCGTGCGCTCTC<br>CTGTTCCGACCCTGCCGCTTACCGGATACCTGTCCGCCTTTCTCCCTTCGGGAAGCGTGGCGCTTTCTCATAGC<br>TCACGCTGTAGGTATCTCAGTTCGGTGTAGGTCGTTCGCTCCAAGCTGGGCTGTGTGCACGAACCCCCCGTTC	Synthesized DNA sequence for GFP expression.

Name	Nucleotide Sequence (5' to 3')	Comment
	AGCCCGACCGCTGCGCCTTATCCGGTAACTATCGTCTTGAGTCCAACCCGGTAAGACACGACTTATCGCCACT<br>GGCAGCAGCCACTGGTAACAGGATTAGCAGAGCGAGGTATGTAGGCGGTGCTACAGAGTTCTTGAAGTGGTG<br>GCCTAACTACGGCTACACTAGAAGAACAGTATTTGGTATCTGCGCTCTGCTGAAGCCAGTTACCTTCGGAAAA<br>AGAGTTGGTAGCTCTTGATCCGGCAAACAAACCACCGCTGGTAGCGGTGGTTTTTTTGTTTGCAAGCAGCAG<br>ATTACGCGCAGAAAAAAAGGATCTCAAGAAGATCCTTTGATCTTTTCTACGGGGTCTGACGCTCAGTGGAAC<br>GAAAACTCACGTTAAGGGATTTTGGTCATGAGATTATCAAAAAGGATCTTCACCTAGATCCTTTTAAATTAAAA<br>ATGAAGTTTTAAATCAATCTAAAGTATATATGAGTAAACTTGGTCTGACAGTTACCAATGCTTAATCAGTGAGG<br>CACCTATCTCAGCGATCTGTCTATTTCGTTCATCCATAGTTGCCTGACTCCCCGTCGTGTAGATAACTACGATAC<br>GGGAGGGCTTACCATCTGGCCCCAGTGCTGCAATGATACCGCGAGAACCACGCTCACCGGCTCCAGATTTATC<br>AGCAATAAACCAGCCAGCCGGAAGGGCCGAGCGCAGAAGTGGTCCTGCAACTTTATCCGCCTCCATCCAGTC<br>TATTAATTGTTGCCGGGAAGCTAGAGTAAGTAGTTCGCCAGTTAATAGTTTGCGCAACGTTGTTGCCATTGCTA<br>CAGGCATCGTGGTGTCACGCTCGTCGTTTGGTATGGCTTCATTCAGCTCCGGTTCCCAACGATCAAGGCGAGT<br>TACATGATCCCCCATGTTGTGCAAAAAAGCGGTTAGCTCCTTCGGTCCTCCGATCGTTGTCAGAAGTAAGTTG<br>GCCGCAGTGTTATCACTCATGGTTATGGCAGCACTGCATAATTCTCTTACTGTCATGCCATCCGTAAGATGCTTT<br>TCTGTGACTGGTGAGTACTCAACCAAGTCATTCTGAGAATAGTGTATGCGGCGACCGAGTTGCTCTTGCCCGG<br>CGTCAATACGGGATAATACCGCGCCACATAGCAGAACTTTAAAAGTGCTCATCATTGGAAAACGTTCTTCGGG<br>GCGAAAACTCTCAAGGATCTTACCGCTGTTGAGATCCAGTTCGATGTAACCCACTCGTGCACCCAACTGATCT<br>TCAGCATCTTTTACTTTCACCAGCGTTTCTGGGTGAGCAAAAACAGGAAGGCAAAATGCCGCAAAAAAGGGA<br>ATAAGGGCGACACGGAAATGTTGAATACTCATACTCTTCCTTTTTCAATATTATTGAAGCATTTATCAGGGTTAT<br>TGTCTCATGAGCGGATACATATTTGAATGTATTTAGAAAAATAAACAAATAGGGGTTCCGCGCACATTTCCCCG<br>AAAAGTGCCACCTGACGTCTAAGAAACCATTATTATCATGACATTAACCTATAAAAATAGGCGTATCACGAGGC<br>CCTTTTGTC	
pUC57-Base64-text	GACAAAAGGGCCTCGTGATACGCCTATTTTTATAGGTTAATGTCATGATAATAATGGTTTCTTAGACGTCAGGTG<br>GCACTTTTCGGGGAAATGTGCGCGGAACCCCTATTTGTTTATTTTTCTAAATACATTCAAATATGTATCCGCTCA<br>TGAGACAATAACCCTGATAAATGCTTCAATAATATTGAAAAAGGAAGAGTATGAGTATTCAACATTTCCGTGTC<br>GCCCTTATTCCCTTTTTTGCGGCATTTTGCCTTCCTGTTTTTGCTCACCCAGAAACGCTGGTGAAAGTAAAAGA<br>TGCTGAAGATCAGTTGGGTGCACGAGTGGGTTACATCGAACTGGATCTCAACAGCGGTAAGATCCTTGAGAG<br>TTTTCGCCCCGAAGAACGTTTTCCAATGATGAGCACTTTTAAAGTTCTGCTATGTGGCGCGGTATTATCCCGTAT<br>TGACGCCGGGCAAGAGCAACTCGGTCGCCGCATACACTATTCTCAGAATGACTTGGTTGAGTACTCACCAGTC<br>ACAGAAAAGCATCTTACGGATGGCATGACAGTAAGAGAATTATGCAGTGCTGCCATAACCATGAGTGATAACA<br>CTGCGGCCAACTTACTTCTGACAACGATCGGAGGACCGAAGGAGCTAACCGCTTTTTTGCACAACATGGGGG<br>ATCATGTAACTCGCCTTGATCGTTGGGAACCGGAGCTGAATGAAGCCATACCAAACGACGAGCGTGACACCA<br>CGATGCCTGTAGCAATGGCAACAACGTTGCGCAAACTATTAACTGGCGAACTACTTACTCTAGCTTCCCGGCA<br>ACAATTAATAGACTGGATGGAGGCGGATAAAGTTGCAGGACCACTTCTGCGCTCGGCCCTTCCGGCTGGCTGG<br>TTTATTGCTGATAAATCTGGAGCCGGTGAGCGTGGTTCTCGCGGTATCATTGCAGCACTGGGGCCAGATGGTA<br>AGCCCTCCCGTATCGTAGTTATCTACACGACGGGGAGTCAGGCAACTATGGATGAACGAAATAGACAGATCGC	Synthesized DNA sequence, which carries the text information encoded by Base64 algorithm. (Addgene ID: 185438)

Name	Nucleotide Sequence (5’ to 3’)	Comment
	TGAGATAGGTGCCTCACTGATTAAGCATTGGTAACTGTCAGACCAAGTTTACTCATATATACTTTAGATTGATTT<br>AAAACTTCATTTTTAATTTAAAAGGATCTAGGTGAAGATCCTTTTTGATAATCTCATGACCAAAATCCCTTAACG<br>TGAGTTTTCGTTCCACTGAGCGTCAGACCCCGTAGAAAAGATCAAAGGATCTTCTTGAGATCCTTTTTTTCTG<br>CGCGTAATCTGCTGCTTGCAAACAAAAAAACCACCGCTACCAGCGGTGGTTTGTTTGCCGGATCAAGAGCTA<br>CCAACTCTTTTTCCGAAGGTAACTGGCTTCAGCAGAGCGCAGATACCAAATACTGTTCTTCTAGTGTAGCCGT<br>AGTTAGGCCACCACTTCAAGAACTCTGTAGCACCGCCTACATACCTCGCTCTGCTAATCCTGTTACCAGTGGCT<br>GCTGCCAGTGGCGATAAGTCGTGTCTTACCGGGTTGGACTCAAGACGATAGTTACCGGATAAGGCGCAGCGG<br>TCGGGCTGAACGGGGGGTTCGTGCACACAGCCCAGCTTGGAGCGAACGACCTACACCGAACTGAGATACCTA<br>CAGCGTGAGCTATGAGAAAGCGCCACGCTTCCCGAAGGGAGAAAGGCGGACAGGTATCCGGTAAGCGGCAG<br>GGTCGGAACAGGAGAGCGCACGAGGGAGCTTCCAGGGGGAAACGCCTGGTATCTTTATAGTCCTGTCGGGTT<br>TCGCCACCTCTGACTTGAGCGTCGATTTTTGTGATGCTCGTCAGGGGGGCGGAGCCTATGGAAAAACGCCAG<br>CAACGCGGCCTTTTTACGGTTCCTGGCCTTTTGCTGGCCTTTTGCTCACATGTTCTTTCCTGCGTTATCCCCTGA<br>TTCTGTGGATAACCGTATTACCGCCTTTGAGTGAGCTGATACCGCTCGCCGCAGCCGAACGACCGAGCGCAGC<br>GAGTCAGTGAGCGAGGAAGCGGAACAGCGCCCAATACGCAAACCGCCTCTCCCCGCGCGTTGGCCGATTCAT<br>TAATGCAGCTGGCACGACAGGTTTCCCGACTGGAAAGCGGGCAGTGAGCGCAACGCAATTAATGTGAGTTAG<br>CTCACTCATTAGGCACCCCAGGCTTTACACTTTATGCTTCCGGCTCGTATGTTGTGTGGAATTGTGAGCGGATA<br>ACAATTTCACACAGGAAACAGCTATGACCATGCTCGAGCCAAGCTCGGCGCGCCATTGGGATGTCGACTTGA<br>CAGCTAGCTCAGTCCTAGGTATAATACTAGTTCGAGATTTTCAGGAGCTAAGGAAGCTAAAGTCTAAGAACTT<br>TAAATAATTTCTACTGTTGTAGATACGAAGTCTGACTCGCAGCCTGTAGGTCTAAGAACTTTAAATAATTTCTAC<br>TGTTGTAGATGAGAAGTCATTTAATAAGGCCACTGGCTCACCTTCGGGTGGGCCTTTCTGCGCAATTGAAGCT<br>T**AGAGAGTACTATAATGCGAGTTACATCTTAAGACTATGATACTTAGctacaggctgcgagtcagacttcgtCAAATTG**<br>**AGAGAATGTCTAAATGTAAGAGATGCTTACAGGACGATGATACATGATGC**GAGCTCTCACTGGAGTTGTC<br>CCAATTCTTGTTGAATTAGATGGTGATGTTAATGGTCACAAATTTTCTGTCAGTGGAGAGGGTGAAGGTGATG<br>CAACATACGGAAAACTTACCCTTAAATTTATTTGCACTACTGGAAAACTACCTGTTCCATGGCCAACACTTGTC<br>ACTACTTTAACTTATGGTGTTCAATGCTTTTCCCGTTATCCGGATCATATGAAACGTCATGACTTTTTCAAGAGT<br>GCCATGCCCGAAGGTTATGTACAGGAACGCACTATATCTTTCAAAGATGACGGTAACTACAAGACGCGTGCTG<br>AAGTCAAGTTTGAAGGTGATACCCTTGTTAATCGTATCGAGTTAAAAGGTATTGATTTTAAAGAAGATGGAAA<br>CATTCTCGGACACAAACTCGAGTACAACTATAACTCACACAATGTATACATCACGGCAGACAAACAAAAGAAT<br>GGAATCAAGGCTAACTTCAAAATTCGCCACAACATTGAAGATGGATCCGTTCAACTAGCAGACCATTATCAAC<br>AAAATACTCCAATTGGCGATGGCCCTGTCCTTTTACCAGACAACCATTACCTGTCGACACAATCTGCCCTTTCG<br>AAAGATCCCAACGAAAAGCGTGACCACATGGTCCTTCTTGAGTTTGTAACTGCTGCTGGTATTACACATGGCA<br>TGGATTAGGAATTCATCCCAATACGCGTCAATTCACTGGCCGTCGTTTTACAACGTCGTGACTGGGAAAACCC<br>TGGCGTTACCCAACTTAATCGCCTTGCAGCACATCCCCCTTTCGCCAGCTGGCGTAATAGCGAAGAGGCCCGC<br>ACCGATCGCCCTTCCCAACAGTTGCGCAGCCTGAATGGCGAATGGCGCCTGATGCGGTATTTTCTCCTTACGC<br>ATCTGTGCGGTATTTCACACCGCATATGGTGCACTCTCAGTACAATCTGCTCTGATGCCGCATAGTTAAGCCAG	

Name	Nucleotide Sequence (5’ to 3’)	Comment
	CCCCGACACCCGCCAACACCCGCTGACGCGCCCTGACGGGCTTGTCTGCTCCCGGCATCCGCTTACAGACAA<br>GCTGTGACAGTCTCCGGGAGCTGCATGTGTCAGAGGTTTTCACCGTCATCACCGAAACGCGCGA	
pUC57-Huffman-text (*ref 45,46*)	TCGCGCGTTTCGGTGATGACGGTGAAAACCTCTGACACATGCAGCTCCCGGAGACTGTCACAGCTTGTCTGT<br>AAGCGGATGCCGGGAGCAGACAAGCCCGTCAGGGCGCGTCAGCGGGTGTTGGCGGGTGTCGGGGCTGGCTT<br>AACTATGCGGCATCAGAGCAGATTGTACTGAGAGTGCACCATATGCGGTGTGAAATACCGCACAGATGCGTAA<br>GGAGAAAATACCGCATCAGGCGCCATTCGCCATTCAGGCTGCGCAACTGTTGGGAAGGGCGATCGGTGCGGG<br>CCTCTTCGCTATTACGCCAGCTGGCGAAAGGGGGATGTGCTGCAAGGCGATTAAGTTGGGTAACGCCAGGGT<br>TTTCCCAGTCACGACGTTGTAAAACGACGGCCAGTGAATTGACGCGTATTGGGATGAATTC**CTATAAGAGCC**<br>**AACTCTTGACCTCCTGGCCACTGGATAGCCCTAACGGCCGT**TTTTA**tcttctgctggtggttcgttcggta**GCTAGCAAA<br>GGAGAAGAACTTTTCACTGGAGTTGTCCCAATTCTTGTTGAATTAGATGGTGATGTTAATGGTCACAAATTTTC<br>TGTCAGTGGAGAGGGTGAAGGTGATGCAACATACGGAAAACTTACCCTTAAATTTATTTGCACTACTGGAAAA<br>CTACCTGTTCCATGGCCAACACTTGTCACTACTTTAACTTATGGTGTTCAATGCTTTTCCCGTTATCCGGATCAT<br>ATGAAACGTCATGACTTTTTCAAGAGTGCCATGCCCGAAGGTTATGTACAGGAACGCACTATATCTTTCAAAG<br>ATGACGGTAACTACAAGACGCGTGCTGAAGTCAAGTTTGAAGGTGATACCCTTGTTAATCGTATCGAGTTAAA<br>AGGTATTGATTTTAAAGAAGATGGAAACATTCTCGGACACAAACTCGAGTACAACTATAACTCACACAATGTA<br>TACATCACGGCAGACAAACAAAAGAATGGAATCAAGGCTAACTTCAAAATTCGCCACAACATTGAAGATGGA<br>TCCGTTCAACTAGCAGACCATTATCAACAAAATACTCCAATTGGCGATGGCCCTGTCCTTTTACCAGACAACC<br>ATTACCTGTCGACACAATCTGCCCTTTCGAAAGATCCCAACGAAAAGCGTGACCACATGGTCCTTCTTGAGTT<br>TGTAACTGCTGCTGGTATTACACATGGCATGGATTAGTTGACAGCTAGCTCAGTCCTAGGTATAATACTAGTTCG<br>AGATTTTCAGGAGCTAAGGAAGCTAAAGTCTAAGAACTTTAAATAATTTCTACTGTTGTAGATTCTTCTGCTGG<br>TGGTTCGTTCGGTAGTCTAAGAACTTTAAATAATTTCTACTGTTGTAGATGAGAAGTCATTTAATAAGGCCACT<br>GGCTCACCTTCGGGTGGGCCTTTCTGCGAAGCTTATCCCAATGGCGCGCCGAGCTTGGCGTAATCATGGTCAT<br>AGCTGTTTCCTGTGTGAAATTGTTATCCGCTCACAATTCCACACAACATACGAGCCGGAAGCATAAAGTGTAA<br>AGCCTGGGGTGCCTAATGAGTGAGCTAACTCACATTAATTGCGTTGCGCTCACTGCCCGCTTTCCAGTCGGGA<br>AACCTGTCGTGCCAGCTGCATTAATGAATCGGCCAACGCGCGGGGAGAGGCGGTTTGCGTATTGGGCGCTGT<br>TCCGCTTCCTCGCTCACTGACTCGCTGCGCTCGGTCGTTCGGCTGCGGCGAGCGGTATCAGCTCACTCAAAGG<br>CGGTAATACGGTTATCCACAGAATCAGGGGATAACGCAGGAAAGAACATGTGAGCAAAAGGCCAGCAAAAG<br>GCCAGGAACCGTAAAAAGGCCGCGTTGCTGGCGTTTTTCCATAGGCTCCGCCCCCCTGACGAGCATCACAAA<br>AATCGACGCTCAAGTCAGAGGTGGCGAAACCCGACAGGACTATAAAGATACCAGGCGTTTCCCCCTGGAAGC<br>TCCCTCGTGCGCTCTCCTGTTCCGACCCTGCCGCTTACCGGATACCTGTCCGCCTTTCTCCCTTCGGGAAGCGT<br>GGCGCTTTCTCATAGCTCACGCTGTAGGTATCTCAGTTCGGTGTAGGTCGTTCGCTCCAAGCTGGGCTGTGTG<br>CACGAACCCCCCGTTCAGCCCGACCGCTGCGCCTTATCCGGTAACTATCGTCTTGAGTCCAACCCGGTAAGAC<br>ACGACTTATCGCCACTGGCAGCAGCCACTGGTAACAGGATTAGCAGAGCGAGGTATGTAGGCGGTGCTACAG<br>AGTTCTTGAAGTGGTGGCCTAACTACGGCTACACTAGAAGAACAGTATTTGGTATCTGCGCTCTGCTGAAGCC<br>AGTTACCTTCGGAAAAAGAGTTGGTAGCTCTTGATCCGGCAAACAAACCACCGCTGGTAGCGGTGGTTTTTTT<br>GTTTGCAAGCAGCAGATTACGCGCAGAAAAAAAGGATCTCAAGAAGATCCTTTGATCTTTTCTACGGGGTCTG	Synthesized DNA sequence, which carries the text information encoded by 15-ary Huffman algorithm. (Addgene ID: 185439)

Name	Nucleotide Sequence (5' to 3')	Comment
	ACGCTCAGTGGAACGAAAACTCACGTTAAGGGATTTTGGTCATGAGATTATCAAAAAGGATCTTCACCTAGAT<br>CCTTTTAAATTAAAAATGAAGTTTTAAATCAATCTAAAGTATATATGAGTAAACTTGGTCTGACAGTTACCAATG<br>CTTAATCAGTGAGGCACCTATCTCAGCGATCTGTCTATTTCGTTCATCCATAGTTGCCTGACTCCCCGTCGTGTA<br>GATAACTACGATACGGGAGGGCTTACCATCTGGCCCCAGTGCTGCAATGATACCGCGAGAACCACGCTCACCG<br>GCTCCAGATTTATCAGCAATAAACCAGCCAGCCGGAAGGGCCGAGCGCAGAAGTGGTCCTGCAACTTTATCC<br>GCCTCCATCCAGTCTATTAATTGTTGCCGGGAAGCTAGAGTAAGTAGTTCGCCAGTTAATAGTTTGCGCAACGT<br>TGTTGCCATTGCTACAGGCATCGTGGTGTCACGCTCGTCGTTTGGTATGGCTTCATTCAGCTCCGGTTCCCAAC<br>GATCAAGGCGAGTTACATGATCCCCCATGTTGTGCAAAAAAGCGGTTAGCTCCTTCGGTCCTCCGATCGTTGT<br>CAGAAGTAAGTTGGCCGCAGTGTTATCACTCATGGTTATGGCAGCACTGCATAATTCTCTTACTGTCATGCCAT<br>CCGTAAGATGCTTTTCTGTGACTGGTGAGTACTCAACCAAGTCATTCTGAGAATAGTGTATGCGGCGACCGAG<br>TTGCTCTTGCCCGGCGTCAATACGGGATAATACCGCGCCACATAGCAGAACTTTAAAAGTGCTCATCATTGGA<br>AAACGTTCTTCGGGGCGAAAACTCTCAAGGATCTTACCGCTGTTGAGATCCAGTTCGATGTAACCCACTCGTG<br>CACCCAACTGATCTTCAGCATCTTTTACTTTCACCAGCGTTTCTGGGTGAGCAAAAACAGGAAGGCAAAATGC<br>CGCAAAAAAGGGAATAAGGGCGACACGGAAATGTTGAATACTCATACTCTTCCTTTTTCAATATTATTGAAGC<br>ATTTATCAGGGTTATTGTCTCATGAGCGGATACATATTTGAATGTATTTAGAAAAATAAACAAATAGGGGTTCCG<br>CGCACATTTCCCCGAAAAGTGCCACCTGACGTCTAAGAAACCATTATTATCATGACATTAACCTATAAAAATAG<br>GCGTATCACGAGGCCCTTTTGTC	
pUC57-Huffman-codebook	TCGCGCGTTTCGGTGATGACGGTGAAAACCTCTGACACATGCAGCTCCCGGAGACTGTCACAGCTTGTCTGT<br>AAGCGGATGCCGGGAGCAGACAAGCCCGTCAGGGCGCGTCAGCGGGTGTTGGCGGGTGTCGGGGCTGGCTT<br>AACTATGCGGCATCAGAGCAGATTGTACTGAGAGTGCACCATATGCGGTGTGAAATACCGCACAGATGCGTAA<br>GGAGAAAATACCGCATCAGGCGCCATTCGCCATTCAGGCTGCGCAACTGTTGGGAAGGGCGATCGGTGCGGG<br>CCTCTTCGCTATTACGCCAGCTGGCGAAAGGGGGATGTGCTGCAAGGCGATTAAGTTGGGTAACGCCAGGGT<br>TTTCCCAGTCACGACGTTGTAAAACGACGGCCAGTGAATTGACGCGTATTGGGATGAATTC**TGTCGTGGGAC**<br>**TCCCAAGTGGTGCGAATCAATTGAATTGGTCTAGGCGATACAAGTCCAACAATTGGAATGACTAGTACT**<br>**TCTAGGCGATACAAGTAGACTACAATTGCTCGCAAATCGTGGGACTCCCAAGACTTGTGA**TTTTA**tcttctgc**<br>**tggtggttcgttcggta**GCTAGCAAAGGAGAAGAACTTTTCACTGGAGTTGTCCCAATTCTTGTTGAATTAGATGGTGA<br>TGTTAATGGTCACAAATTTTCTGTCAGTGGAGAGGGTGAAGGTGATGCAACATACGGAAAACTTACCCTTAAA<br>TTTATTTGCACTACTGGAAAACTACCTGTTCCATGGCCAACACTTGTCACTACTTTAACTTATGGTGTTCAATGC<br>TTTTCCCGTTATCCGGATCATATGAAACGTCATGACTTTTTCAAGAGTGCCATGCCCGAAGGTTATGTACAGGA<br>ACGCACTATATCTTTCAAAGATGACGGTAACTACAAGACGCGTGCTGAAGTCAAGTTTGAAGGTGATACCCTT<br>GTTAATCGTATCGAGTTAAAAGGTATTGATTTTAAAGAAGATGGAAACATTCTCGGACACAAACTCGAGTACA<br>ACTATAACTCACACAATGTATACATCACGGCAGACAAACAAAAGAATGGAATCAAGGCTAACTTCAAAATTCG<br>CCACAACATTGAAGATGGATCCGTTCAACTAGCAGACCATTATCAACAAAATACTCCAATTGGCGATGGCCCT<br>GTCCTTTTACCAGACAACCATTACCTGTCGACACAATCTGCCCTTTCGAAAGATCCCAACGAAAAGCGTGACC<br>ACATGGTCCTTCTTGAGTTTGTAACTGCTGCTGGTATTACACATGGCATGGATTAGTTGACAGCTAGCTCAGTC<br>CTAGGTATAATACTAGTTCGAGATTTTCAGGAGCTAAGGAAGCTAAAGTCTAAGAACTTTAAATAATTTCTACT	Synthesized DNA sequence, which carries the codebook information encoded by 15-ary Huffman algorithm. (Addgene ID: 185440)

Name	Nucleotide Sequence (5’ to 3’)	Comment
	GTTGTAGATTCTTCTGCTGGTGGTTCGTTCGGTAGTCTAAGAACTTTAAATAATTTCTACTGTTGTAGATGAGA<br>AGTCATTTAATAAGGCCACTGGCTCACCTTCGGGTGGGCCTTTCTGCGAAGCTTATCCCAATGGCGCGCCGAG<br>CTTGGCGTAATCATGGTCATAGCTGTTTCCTGTGTGAAATTGTTATCCGCTCACAATTCCACACAACATACGAG<br>CCGGAAGCATAAAGTGTAAAGCCTGGGGTGCCTAATGAGTGAGCTAACTCACATTAATTGCGTTGCGCTCACT<br>GCCCGCTTTCCAGTCGGGAAACCTGTCGTGCCAGCTGCATTAATGAATCGGCCAACGCGCGGGGAGAGGCGG<br>TTTGCGTATTGGGCGCTGTTCCGCTTCCTCGCTCACTGACTCGCTGCGCTCGGTCGTTCGGCTGCGGCGAGCG<br>GTATCAGCTCACTCAAAGGCGGTAATACGGTTATCCACAGAATCAGGGGATAACGCAGGAAAGAACATGTGA<br>GCAAAAGGCCAGCAAAAGGCCAGGAACCGTAAAAAGGCCGCGTTGCTGGCGTTTTTCCATAGGCTCCGCCC<br>CCCTGACGAGCATCACAAAAATCGACGCTCAAGTCAGAGGTGGCGAAACCCGACAGGACTATAAAGATACCA<br>GGCGTTTCCCCCTGGAAGCTCCCTCGTGCGCTCTCCTGTTCCGACCCTGCCGCTTACCGGATACCTGTCCGCC<br>TTTCTCCCTTCGGGAAGCGTGGCGCTTTCTCATAGCTCACGCTGTAGGTATCTCAGTTCGGTGTAGGTCGTTCG<br>CTCCAAGCTGGGCTGTGTGCACGAACCCCCCGTTCAGCCCGACCGCTGCGCCTTATCCGGTAACTATCGTCTT<br>GAGTCCAACCCGGTAAGACACGACTTATCGCCACTGGCAGCAGCCACTGGTAACAGGATTAGCAGAGCGAGG<br>TATGTAGGCGGTGCTACAGAGTTCTTGAAGTGGTGGCCTAACTACGGCTACACTAGAAGAACAGTATTTGGTA<br>TCTGCGCTCTGCTGAAGCCAGTTACCTTCGGAAAAAGAGTTGGTAGCTCTTGATCCGGCAAACAAACCACCG<br>CTGGTAGCGGTGGTTTTTTTGTTTGCAAGCAGCAGATTACGCGCAGAAAAAAAGGATCTCAAGAAGATCCTTT<br>GATCTTTTCTACGGGGTCTGACGCTCAGTGGAACGAAAACTCACGTTAAGGGATTTTGGTCATGAGATTATCA<br>AAAAGGATCTTCACCTAGATCCTTTTAAATTAAAAATGAAGTTTTAAATCAATCTAAAGTATATATGAGTAAACT<br>TGGTCTGACAGTTACCAATGCTTAATCAGTGAGGCACCTATCTCAGCGATCTGTCTATTTCGTTCATCCATAGTT<br>GCCTGACTCCCCGTCGTGTAGATAACTACGATACGGGAGGGCTTACCATCTGGCCCCAGTGCTGCAATGATAC<br>CGCGAGAACCACGCTCACCGGCTCCAGATTTATCAGCAATAAACCAGCCAGCCGGAAGGGCCGAGCGCAGA<br>AGTGGTCCTGCAACTTTATCCGCCTCCATCCAGTCTATTAATTGTTGCCGGGAAGCTAGAGTAAGTAGTTCGCC<br>AGTTAATAGTTTGCGCAACGTTGTTGCCATTGCTACAGGCATCGTGGTGTCACGCTCGTCGTTTGGTATGGCTT<br>CATTCAGCTCCGGTTCCCAACGATCAAGGCGAGTTACATGATCCCCCATGTTGTGCAAAAAAGCGGTTAGCTC<br>CTTCGGTCCTCCGATCGTTGTCAGAAGTAAGTTGGCCGCAGTGTTATCACTCATGGTTATGGCAGCACTGCATA<br>ATTCTCTTACTGTCATGCCATCCGTAAGATGCTTTTCTGTGACTGGTGAGTACTCAACCAAGTCATTCTGAGAA<br>TAGTGTATGCGGCGACCGAGTTGCTCTTGCCCGGCGTCAATACGGGATAATACCGCGCCACATAGCAGAACTT<br>TAAAAGTGCTCATCATTGGAAAACGTTCTTCGGGGCGAAAACTCTCAAGGATCTTACCGCTGTTGAGATCCAG<br>TTCGATGTAACCCACTCGTGCACCCAACTGATCTTCAGCATCTTTTACTTTCACCAGCGTTTCTGGGTGAGCAA<br>AAACAGGAAGGCAAAATGCCGCAAAAAAGGGAATAAGGGCGACACGGAAATGTTGAATACTCATACTCTTCC<br>TTTTTCAATATTATTGAAGCATTTATCAGGGTTATTGTCTCATGAGCGGATACATATTTGAATGTATTTAGAAAAA<br>TAAACAAATAGGGGTTCCGCGCACATTTCCCCGAAAAGTGCCACCTGACGTCTAAGAAACCATTATTATCATG<br>ACATTAACCTATAAAAATAGGCGTATCACGAGGCCCTTTTGTC	
pUC57-Huffman-image	TCGCGCGTTTCGGTGATGACGGTGAAAACCTCTGACACATGCAGCTCCCGGAGACTGTCACAGCTTGTCTGT<br>AAGCGGATGCCGGGAGCAGACAAGCCCGTCAGGGCGCGTCAGCGGGTGTTGGCGGGTGTCGGGGCTGGCTT<br>AACTATGCGGCATCAGAGCAGATTGTACTGAGAGTGCACCATATGCGGTGTGAAATACCGCACAGATGCGTAA	Synthesized DNA sequence, which carries the

Name	Nucleotide Sequence (5' to 3')	Comment
	GGAGAAAATACCGCATCAGGCGCCATTCGCCATTCAGGCTGCGCAACTGTTGGGAAGGGCGATCGGTGCGGG<br>CCTCTTCGCTATTACGCCAGCTGGCGAAAGGGGGATGTGCTGCAAGGCGATTAAGTTGGGTAACGCCAGGGT<br>TTTCCCAGTCACGACGTTGTAAAACGACGGCCAGTGAATTGACGCGTATTGGGATGAATTC**ACATCGTCGGC<br>TCAACTGGTTTTAGCCGGTGACCAGGGCTCAACGCCGATAACTCAGGCTCAACTGGTGACCAGTTTAG<br>CCGATAATCGGCTCAACTGGTGACCAGTTGTCTATAATCGGCTCAACTGGTGACCAGTTTAGCCGATAA<br>TCGGCTCAAATAGACAAATAGCCGATAATCGGCTCATCCTAGGTCCAAGCATTGCTACTACCTTGGCTC<br>AACTGGTGACCAGTTTAGCCGATAAGCATTGCTACTACCTTGGATTCGAACGTCGTTGCCATCCTAGGT<br>CCAATCGGCTCAACTGGTGAACGTCGTTGCCATCCTAGGTCCAAGCATTGCTACTACCTTGGATTCGAC<br>CAGTTTAGCCGATAAGCATTGCTACTACCTTGGCTCAACTGGTGAACGTCGTTGCCATCCTAGTTTAGC<br>CGATAATCGGATTCGAACGTCGATAATCGGCTCAACTGGTGACCAGGTCCAAGCATTGGTGACCAGTT<br>TAGCCGATAATCCGAAGTCATGGCAGTACTCAACTGCTACTACCTTGGCTCAACTGGTGACCAGTGGG<br>CGATAATCGGCTCAACTGGTGACCAGTTTAGCCGATAATCGGCTCAACTGGGCGAGTTTAGACAAATAG<br>ACAAATAGACAAATAGACAAATCGGCTATTTGTCTATTTGTCTATTTGTCTCAACTCGCCCACTCGCCCA<br>CTCGCCCACTCGCCCAGTTTAGCCGATTTGTCTATTTGTCTATTTTAGC**TTTTA**tcttctgctggtggttcgttcggta**GC<br>TAGCAAAGGAGAAGAACTTTTCACTGGAGTTGTCCCAATTCTTGTTGAATTAGATGGTGATGTTAATGGTCAC<br>AAATTTTCTGTCAGTGGAGAGGGTGAAGGTGATGCAACATACGGAAAACTTACCCTTAAATTTATTTGCACTA<br>CTGGAAAACTACCTGTTCCATGGCCAACACTTGTCACTACTTTAACTTATGGTGTTCAATGCTTTTCCCGTTATC<br>CGGATCATATGAAACGTCATGACTTTTTCAAGAGTGCCATGCCCGAAGGTTATGTACAGGAACGCACTATATCT<br>TTCAAAGATGACGGTAACTACAAGACGCGTGCTGAAGTCAAGTTTGAAGGTGATACCCTTGTTAATCGTATCG<br>AGTTAAAAGGTATTGATTTTAAAGAAGATGGAAACATTCTCGGACACAAACTCGAGTACAACTATAACTCACA<br>CAATGTATACATCACGGCAGACAAACAAAAGAATGGAATCAAGGCTAACTTCAAAATTCGCCACAACATTGA<br>AGATGGATCCGTTCAACTAGCAGACCATTATCAACAAAATACTCCAATTGGCGATGGCCCTGTCCTTTTACCAG<br>ACAACCATTACCTGTCGACACAATCTGCCCTTTCGAAAGATCCCAACGAAAAGCGTGACCACATGGTCCTTCT<br>TGAGTTTGTAACTGCTGCTGGTATTACACATGGCATGGATTAGTTGACAGCTAGCTCAGTCCTAGGTATAATAC<br>TAGTTCGAGATTTCAGGAGCTAAGGAAGCTAAAGTCTAAGAACTTTAAATAATTTCTACTGTTGTAGATTCTT<br>CTGCTGGTGGTTCGTTCGGTAGTCTAAGAACTTTAAATAATTTCTACTGTTGTAGATGAGAAGTCATTTAATAA<br>GGCCACTGGCTCACCTTCGGGTGGGCCTTTCTGCGAAGCTTATCCCAATGGCGCGCCGAGCTTGGCGTAATCA<br>TGGTCATAGCTGTTTCCTGTGTGAAATTGTTATCCGCTCACAATTCCACACAACATACGAGCCGGAAGCATAAA<br>GTGTAAAGCCTGGGGTGCCTAATGAGTGAGCTAACTCACATTAATTGCGTTGCGCTCACTGCCCGCTTTCCAG<br>TCGGGAAACCTGTCGTGCCAGCTGCATTAATGAATCGGCCAACGCGCGGGGAGAGGCGGTTTGCGTATTGGG<br>CGCTGTTCCGCTTCCTCGCTCACTGACTCGCTGCGCTCGGTCGTTCGGCTGCGGCGAGCGGTATCAGCTCACT<br>CAAAGGCGGTAATACGGTTATCCACAGAATCAGGGGATAACGCAGGAAAGAACATGTGAGCAAAAGGCCAG<br>CAAAAGGCCAGGAACCGTAAAAAGGCCGCGTTGCTGGCGTTTTTCCATAGGCTCCGCCCCCCTGACGAGCAT<br>CACAAAAATCGACGCTCAAGTCAGAGGTGGCGAAACCCGACAGGACTATAAAGATACCAGGCGTTTCCCCCT<br>GGAAGCTCCCTCGTGCGCTCTCCTGTTCCGACCCTGCCGCTTACCGGATACCTGTCCGCCTTTCTCCCTTCGGG<br>AAGCGTGGCGCTTTCTCATAGCTCACGCTGTAGGTATCTCAGTTCGGTGTAGGTCGTTCGCTCCAAGCTGGGC	image information encoded by 15-ary Huffman algorithm. (Addgene ID: 185441)

Name	Nucleotide Sequence (5' to 3')	Comment
	TGTGTGCACGAACCCCCCGTTCAGCCCGACCGCTGCGCCTTATCCGGTAACTATCGTCTTGAGTCCAACCCGG<br>TAAGACACGACTTATCGCCACTGGCAGCAGCCACTGGTAACAGGATTAGCAGAGCGAGGTATGTAGGCGGTG<br>CTACAGAGTTCTTGAAGTGGTGGCCTAACTACGGCTACACTAGAAGAACAGTATTTGGTATCTGCGCTCTGCT<br>GAAGCCAGTTACCTTCGGAAAAAGAGTTGGTAGCTCTTGATCCGGCAAACAAACCACCGCTGGTAGCGGTGG<br>TTTTTTTGTTTGCAAGCAGCAGATTACGCGCAGAAAAAAAGGATCTCAAGAAGATCCTTTGATCTTTTCTACG<br>GGGTCTGACGCTCAGTGGAACGAAAACTCACGTTAAGGGATTTTGGTCATGAGATTATCAAAAAGGATCTTCA<br>CCTAGATCCTTTTAAATTAAAAATGAAGTTTTAAATCAATCTAAAGTATATATGAGTAAACTTGGTCTGACAGTT<br>ACCAATGCTTAATCAGTGAGGCACCTATCTCAGCGATCTGTCTATTTCGTTCATCCATAGTTGCCTGACTCCCCG<br>TCGTGTAGATAACTACGATACGGGAGGGCTTACCATCTGGCCCCAGTGCTGCAATGATACCGCGAGAACCACG<br>CTCACCGGCTCCAGATTTATCAGCAATAAACCAGCCAGCCGGAAGGGCCGAGCGCAGAAGTGGTCCTGCAAC<br>TTTATCCGCCTCCATCCAGTCTATTAATTGTTGCCGGGAAGCTAGAGTAAGTAGTTCGCCAGTTAATAGTTTGC<br>GCAACGTTGTTGCCATTGCTACAGGCATCGTGGTGTCACGCTCGTCGTTTGGTATGGCTTCATTCAGCTCCGGT<br>TCCCAACGATCAAGGCGAGTTACATGATCCCCCATGTTGTGCAAAAAAGCGGTTAGCTCCTTCGGTCCTCCGA<br>TCGTTGTCAGAAGTAAGTTGGCCGCAGTGTTATCACTCATGGTTATGGCAGCACTGCATAATTCTCTTACTGTC<br>ATGCCATCCGTAAGATGCTTTTCTGTGACTGGTGAGTACTCAACCAAGTCATTCTGAGAATAGTGTATGCGGCG<br>ACCGAGTTGCTCTTGCCCGGCGTCAATACGGGATAATACCGCGCCACATAGCAGAACTTTAAAAGTGCTCATC<br>ATTGGAAAACGTTCTTCGGGGCGAAAACTCTCAAGGATCTTACCGCTGTTGAGATCCAGTTCGATGTAACCCA<br>CTCGTGCACCCAACTGATCTTCAGCATCTTTTACTTTCACCAGCGTTTCTGGGTGAGCAAAAACAGGAAGGCA<br>AAATGCCGCAAAAAAGGGAATAAGGGCGACACGGAAATGTTGAATACTCATACTCTTCCTTTTTCAATATTATT<br>GAAGCATTTATCAGGGTTATTGTCTCATGAGCGGATACATATTTGAATGTATTTAGAAAAATAAACAAATAGGG<br>GTTCCGCGCACATTTCCCCGAAAAGTGCCACCTGACGTCTAAGAAACCATTATTATCATGACATTAACCTATAA<br>AAATAGGCGTATCACGAGGCCCTTTGTC	
pUC57-newimage	TCGCGCGTTTCGGTGATGACGGTGAAAACCTCTGACACATGCAGCTCCCGGAGACTGTCACAGCTTGTCTGT<br>AAGCGGATGCCGGGAGCAGACAAGCCCGTCAGGGCGCGTCAGCGGGTGTTGGCGGGTGTCGGGGCTGGCTT<br>AACTATGCGGCATCAGAGCAGATTGTACTGAGAGTGCACCATATGCGGTGTGAAATACCGCACAGATGCGTAA<br>GGAGAAAATACCGCATCAGGCGCCATTCGCCATTCAGGCTGCGCAACTGTTGGGAAGGGCGATCGGTGCGGG<br>CCTCTTCGCTATTACGCCAGCTGGCGAAAGGGGGATGTGCTGCAAGGCGATTAAGTTGGGTAACGCCAGGGT<br>TTTCCCAGTCACGACGTTGTAAAACGACGGCCAGTGAATTGACGCGTATTGGGATGAATTC**ACATAGGCGAC**<br>**TCGCCCAATCGCCCAATCTCGCCCATAGTGGGCACAATTTGCCCAGCGACTCGCCCAGGGCGACTAAG**<br>**GGCGAACGTGGGCGACTCGCCCAATAGACTAGACTCGCCCAATAGACAATTTGTCTAGTGGCCACTAG**<br>**ACTCGCCCAACTTTAGGCTCCAGAATCGTGGGCGACTCGCCCAATAATGGTGCCGCAACTTTAGGCTC**<br>**CAGAATCGTGGGCGACTCAAGTTTCGGGACCATAATGGTGCCGCAACTTTGTCTAGTGACGATACTGT**<br>**AGTGGGCGACCATAATGGTGCGACTCGCCAGAATCGTGGGCGACTCGCCCAATAATGGTGCGACTCGC**<br>**CCAATAATGGTGCCGCAATAGTTTCGGGACTCGCCCATTGTGGGCGACTCGCCCAATAGACAATTTGTC**<br>**TAGTCGAGTCGCCAAAGTCTAGTGGCCCCAATTGGGCCACTAGAATTTGGCGACTCGACATTGTCGAG**<br>**ACAATTTGGCCCAATACTATAGAC**AAGCTTATCCCAATGGCGCGCCGAGCTTGGCGTAATCATGGTCATAGCT	Synthesized plasmid containing rewritten image information of DNA sequence encoded by 15-ary Huffman algorithm.

Name	Nucleotide Sequence (5' to 3')	Comment
	GTTTCCTGTGTGAAATTGTTATCCGCTCACAATTCCACACAACATACGAGCCGGAAGCATAAAGTGTAAAGCC<br>TGGGGTGCCTAATGAGTGAGCTAACTCACATTAATTGCGTTGCGCTCACTGCCCGCTTTCCAGTCGGGAAACC<br>TGTCGTGCCAGCTGCATTAATGAATCGGCCAACGCGCGGGGAGAGGCGGTTTGCGTATTGGGCGCTGTTCCGC<br>TTCCTCGCTCACTGACTCGCTGCGCTCGGTCGTTCGGCTGCGGCGAGCGGTATCAGCTCACTCAAAGGCGGTA<br>ATACGGTTATCCACAGAATCAGGGGATAACGCAGGAAAGAACATGTGAGCAAAAGGCCAGCAAAAGGCCAG<br>GAACCGTAAAAAGGCCGCGTTGCTGGCGTTTTTCCATAGGCTCCGCCCCCCTGACGAGCATCACAAAAATCG<br>ACGCTCAAGTCAGAGGTGGCGAAACCCGACAGGACTATAAAGATACCAGGCGTTTCCCCCTGGAAGCTCCCT<br>CGTGCGCTCTCCTGTTCCGACCCTGCCGCTTACCGGATACCTGTCCGCCTTTCTCCCTTCGGGAAGCGTGGCG<br>CTTTCTCATAGCTCACGCTGTAGGTATCTCAGTTCGGTGTAGGTCGTTCGCTCCAAGCTGGGCTGTGTGCACG<br>AACCCCCCGTTCAGCCCGACCGCTGCGCCTTATCCGGTAACTATCGTCTTGAGTCCAACCCGGTAAGACACGA<br>CTTATCGCCACTGGCAGCAGCCACTGGTAACAGGATTAGCAGAGCGAGGTATGTAGGCGGTGCTACAGAGTT<br>CTTGAAGTGGTGGCCTAACTACGGCTACACTAGAAGAACAGTATTTGGTATCTGCGCTCTGCTGAAGCCAGTT<br>ACCTTCGGAAAAAGAGTTGGTAGCTCTTGATCCGGCAAACAAACCACCGCTGGTAGCGGTGGTTTTTTTGTTT<br>GCAAGCAGCAGATTACGCGCAGAAAAAAAGGATCTCAAGAAGATCCTTTGATCTTTTCTACGGGGTCTGACG<br>CTCAGTGGAACGAAAACTCACGTTAAGGGATTTTGGTCATGAGATTATCAAAAAGGATCTTCACCTAGATCCT<br>TTTAAATTAAAAATGAAGTTTTAAATCAATCTAAAGTATATATGAGTAAACTTGGTCTGACAGTTACCAATGCTT<br>AATCAGTGAGGCACCTATCTCAGCGATCTGTCTATTTCGTTCATCCATAGTTGCCTGACTCCCCGTCGTGTAGAT<br>AACTACGATACGGGAGGGCTTACCATCTGGCCCCAGTGCTGCAATGATACCGCGAGAACCACGCTCACCGGC<br>TCCAGATTTATCAGCAATAAACCAGCCAGCCGGAAGGGCCGAGCGCAGAAGTGGTCCTGCAACTTTATCCGC<br>CTCCATCCAGTCTATTAATTGTTGCCGGGAAGCTAGAGTAAGTAGTTCGCCAGTTAATAGTTTGCGCAACGTTG<br>TTGCCATTGCTACAGGCATCGTGGTGTCACGCTCGTCGTTTGGTATGGCTTCATTCAGCTCCGGTTCCCAACGA<br>TCAAGGCGAGTTACATGATCCCCCATGTTGTGCAAAAAAGCGGTTAGCTCCTTCGGTCCTCCGATCGTTGTCA<br>GAAGTAAGTTGGCCGCAGTGTTATCACTCATGGTTATGGCAGCACTGCATAATTCTCTTACTGTCATGCCATCC<br>GTAAGATGCTTTTCTGTGACTGGTGAGTACTCAACCAAGTCATTCTGAGAATAGTGTATGCGGCGACCGAGTT<br>GCTCTTGCCCGGCGTCAATACGGGATAATACCGCGCCACATAGCAGAACTTTAAAAGTGCTCATCATTGGAAA<br>ACGTTCTTCGGGGCGAAAACTCTCAAGGATCTTACCGCTGTTGAGATCCAGTTCGATGTAACCCACTCGTGCA<br>CCCAACTGATCTTCAGCATCTTTTACTTTCACCAGCGTTTCTGGGTGAGCAAAAACAGGAAGGCAAAATGCCG<br>CAAAAAAGGGAATAAGGGCGACACGGAAATGTTGAATACTCATACTCTTCCTTTTTCAATATTATTGAAGCATT<br>TATCAGGGTTATTGTCTCATGAGCGGATACATATTTGAATGTATTTAGAAAAATAAACAAATAGGGGTTCCGCG<br>CACATTTCCCCGAAAAGTGCCACCTGACGTCTAAGAAACCATTATTATCATGACATTAACCTATAAAAATAGGC<br>GTATCACGAGGCCCTTTGTC	
Base64-newtext (donor DNA)	TGGAAAAACGCCAGCAACGCGGCCTTTTTACGGTTCCTGGCCTTTTGCTGGCCTTTTGCTCACATGTTCTTTCC<br>TGCGTTATCCCCTGATTCTGTGGATAACCGTATTACCGCCTTTGAGTGAGCTGATACCGCTCGCCGCAGCCGAA<br>CGACCGAGCGCAGCGAGTCAGTGAGCGAGGAAGCGGAACAGCGCCCAATACGCAAACCGCCTCTCCCCGCG<br>CGTTGGCCGATTCATTAATGCAGCTGGCACGACAGGTTTCCCGACTGGAAAGCGGGCAGTGAGCGCAACGCA<br>ATTAATGTGAGTTAGCTCACTCATTAGGCACCCCAGGCTTTACACTTTATGCTTCCGGCTCGTATGTTGTGTGG	Donor DNA sequence including rewritten DNA fragment and

Name	Nucleotide Sequence (5’ to 3’)	Comment
	AATTGTGAGCGGATAACAATTTCACACAGGAAACAGCTATGACCATGCTCGAGCCAAGCTCGGCGCGCCATT<br>GGGATAAGCTT**AGAGAGTACTATAATGCGAGTTACATCTTAAGACTATGATACTTAGCTAAATGATTAGAG**<br>**AGCTAGACTATTCCGCTCTTAAGATTGAACTATGAGAGAGTACTAGATGGCACATTTATATAGTATGC**GA<br>GCTCCGATCCCGCGAAATTAATACGAGCTGTTGACAATTAATCATCGGCTCGTATAATGTGTGGAATTGTGAGC<br>GGATAACAATTCCCCTCTAGAAATAATTTTGTTTAACTTTAAGAAGGAGATATACATATGGCTAGCAAAGGAGA<br>AGAACTTTTCACTGGAGTTGTCCCAATTCTTGTTGAATTAGATGGTGATGTTAATGGTCACAAATTTTCTGTCA<br>GTGGAGAGGGTGAAGGTGATGCAACATACGGAAAACTTACCCTTAAATTTATTTGCACTACTGGAAAACTACC<br>TGTTCCATGGCCAACACTTGTCACTACTTTAACTTATGGTGTTCAATGCTTTTCCCGTTATCCGGATCATATGAA<br>ACGTCATGACTTTTTCAAGAGTGCCATGCCCGAAGGTTATGTACAGGAACGCACTATATCTTTCAAAGATGAC<br>GGTAACTACAAGACGCGTGCTGAAGTCAAGTTTGAAGGTGATACCCTTGTTAATCGTATCGAGTTAAAAGGTA<br>TTGATTTTAAAGAAGATGGAAACATTCTCGGACACAAACTCGAGTACAACTATAACTCACACAATGTATACATC<br>ACGGCAGA	two 500 bp homologous arms.
Huffman-newtext (donor DNA, *ref.47*)	CGTTTCGGTGATGACGGTGAAAACCTCTGACACATGCAGCTCCCGGAGACTGTCACAGCTTGTCTGTAAGCG<br>GATGCCGGGAGCAGACAAGCCCGTCAGGGCGCGTCAGCGGGTGTTGGCGGGTGTCGGGGCTGGCTTAACTAT<br>GCGGCATCAGAGCAGATTGTACTGAGAGTGCACCATATGCGGTGTGAAATACCGCACAGATGCGTAAGGAGA<br>AAATACCGCATCAGGCGCCATTCGCCATTCAGGCTGCGCAACTGTTGGGAAGGGCGATCGGTGCGGGCCTCTT<br>CGCTATTACGCCAGCTGGCGAAAGGGGGATGTGCTGCAAGGCGATTAAGTTGGGTAACGCCAGGGTTTTCCC<br>AGTCACGACGTTGTAAAACGACGGCCAGTGAATTGACGCGTATTGGGATGAATTC**GCCCAGATCCTTTAGAG**<br>**TCGATCTAGTAGACCGGAATCGCAGGCCATGCG**CGATCCCGCGAAATTAATACGAGCTGTTGACAATTAAT<br>CATCGGCTCGTATAATGTGTGGAATTGTGAGCGGATAACAATTCCCCTCTAGAAATAATTTTGTTTAACTTTAAG<br>AAGGAGATATACATATGGCTAGCAAAGGAGAAGAACTTTTCACTGGAGTTGTCCCAATTCTTGTTGAATTAGAT<br>GGTGATGTTAATGGTCACAAATTTTCTGTCAGTGGAGAGGGTGAAGGTGATGCAACATACGGAAAACTTACCC<br>TTAAATTTATTTGCACTACTGGAAAACTACCTGTTCCATGGCCAACACTTGTCACTACTTTAACTTATGGTGTTC<br>AATGCTTTTCCCGTTATCCGGATCATATGAAACGTCATGACTTTTTCAAGAGTGCCATGCCCGAAGGTTATGTA<br>CAGGAACGCACTATATCTTTCAAAGATGACGGTAACTACAAGACGCGTGCTGAAGTCAAGTTTGAAGGTGATA<br>CCCTTGTTAATCGTATCGAGTTAAAAGGTATTGATTTTAAAGAAGATGGAAACATTCTCGGACACAAACTCGA<br>GTACAACTATAACTCACACAATGTATACATCACGGCAGA	Donor DNA sequence including rewritten DNA fragment and two 500 bp homologous arms.
Huffman-newcodebook (donor DNA)	CGTTTCGGTGATGACGGTGAAAACCTCTGACACATGCAGCTCCCGGAGACTGTCACAGCTTGTCTGTAAGCG<br>GATGCCGGGAGCAGACAAGCCCGTCAGGGCGCGTCAGCGGGTGTTGGCGGGTGTCGGGGCTGGCTTAACTAT<br>GCGGCATCAGAGCAGATTGTACTGAGAGTGCACCATATGCGGTGTGAAATACCGCACAGATGCGTAAGGAGA<br>AAATACCGCATCAGGCGCCATTCGCCATTCAGGCTGCGCAACTGTTGGGAAGGGCGATCGGTGCGGGCCTCTT<br>CGCTATTACGCCAGCTGGCGAAAGGGGGATGTGCTGCAAGGCGATTAAGTTGGGTAACGCCAGGGTTTTCCC<br>AGTCACGACGTTGTAAAACGACGGCCAGTGAATTGACGCGTATTGGGATGAATTC**TGTCGTGGGACTCCCA**<br>**AGTGGTGCGAATCAATTGAATTGGTCTAGGCGATACAAGTTTTGGCTGTCGATGTCGTCTCAAGACTAC**<br>**GACGACTAGTACTTTTGGCATTGCTCGCAAATCGTGGGACTCCCAAGACTTGTGA**CGATCCCGCGAAAT	Donor DNA sequences include rewritten DNA fragment and two 500 bp arms, which were consistent with the upstream and

Name	Nucleotide Sequence (5’ to 3’)	Comment
	TAATACGAGCTGTTGACAATTAATCATCGGCTCGTATAATGTGTGGAATTGTGAGCGGATAACAATTCCCCTCTA<br>GAAATAATTTTGTTTAACTTTAAGAAGGAGATATACATATGGCTAGCAAAGGAGAAGAACTTTTCACTGGAGTT<br>GTCCCAATTCTTGTTGAATTAGATGGTGATGTTAATGGTCACAAATTTTCTGTCAGTGGAGAGGGTGAAGGTG<br>ATGCAACATACGGAAAACTTACCCTTAAATTTATTTGCACTACTGGAAAACTACCTGTTCCATGGCCAACACTT<br>GTCACTACTTTAACTTATGGTGTTCAATGCTTTTCCCGTTATCCGGATCATATGAAACGTCATGACTTTTTCAAG<br>AGTGCCATGCCCGAAGGTTATGTACAGGAACGCACTATATCTTTCAAAGATGACGGTAACTACAAGACGCGTG<br>CTGAAGTCAAGTTTGAAGGTGATACCCTTGTTAATCGTATCGAGTTAAAAGGTATTGATTTTAAAGAAGATGGA<br>AACATTCTCGGACACAAACTCGAGTACAACTATAACTCACACAATGTATACATCACGGCAGA	downstream DNA sequences of rewritten DNA fragment.
Huffman-newimage donor DNA	CGTTTCGGTGATGACGGTGAAAACCTCTGACACATGCAGCTCCCGGAGACTGTCACAGCTTGTCTGTAAGCG<br>GATGCCGGGAGCAGACAAGCCCGTCAGGGCGCGTCAGCGGGTGTTGGCGGGTGTCGGGGCTGGCTTAACTAT<br>GCGGCATCAGAGCAGATTGTACTGAGAGTGCACCATATGCGGTGTGAAATACCGCACAGATGCGTAAGGAGA<br>AAATACCGCATCAGGCGCCATTCGCCATTCAGGCTGCGCAACTGTTGGGAAGGGCGATCGGTGCGGGCCTCTT<br>CGCTATTACGCCAGCTGGCGAAAGGGGGATGTGCTGCAAGGCGATTAAGTTGGGTAACGCCAGGGTTTTCCC<br>AGTCACGACGTTGTAAAACGACGGCCAGTGAATTGACGCGTATTGGGATGGAACGAATTC**ACATAGGCGACT**<br>**CGCCCAATCGCCCAATCTCGCCCATAGTGGGCACAATTTGCCCAGCGACTCGCCCAGGGCGACTAAGG**<br>**GCGAACGTGGGCGACTCGCCCAATAGACTAGACTCGCCCAATAGACAATTTGTCTAGTGGCCACTAGA**<br>**CTCGCCCAACTTTAGGCTCCAGAATCGTGGGCGACTCGCCCAATAATGGTGCCGCAACTTTAGGCTCC**<br>**AGAATCGTGGGCGACTCAAGTTTCGGGACCATAATGGTGCCGCAACTTTGTCTAGTGACGATACTGTA**<br>**GTGGGCGACCATAATGGTGCGACTCGCCAGAATCGTGGGCGACTCGCCCAATAATGGTGCGACTCGCC**<br>**CAATAATGGTGCCGCAATAGTTTCGGGACTCGCCCATTGTGGGCGACTCGCCCAATAGACAATTTGTCT**<br>**AGTCGAGTCGCCAAAGTCTAGTGGCCCCAATTGGGCCACTAGAATTTGGCGACTCGACATTGTCGAGA**<br>**CAATTTGGCCCAATACTATAGAC**CGATCCCGCGAAATTAATACGAGCTGTTGACAATTAATCATCGGCTCGTA<br>TAATGTGTGGAATTGTGAGCGGATAACAATTCCCCTCTAGAAATAATTTTGTTTAACTTTAAGAAGGAGATATA<br>CATATGGCTAGCAAAGGAGAAGAACTTTTCACTGGAGTTGTCCCAATTCTTGTTGAATTAGATGGTGATGTTAA<br>TGGTCACAAATTTTCTGTCAGTGGAGAGGGTGAAGGTGATGCAACATACGGAAAACTTACCCTTAAATTTATT<br>TGCACTACTGGAAAACTACCTGTTCCATGGCCAACACTTGTCACTACTTTAACTTATGGTGTTCAATGCTTTTC<br>CCGTTATCCGGATCATATGAAACGTCATGACTTTTTCAAGAGTGCCATGCCCGAAGGTTATGTACAGGAACGC<br>ACTATATCTTTCAAAGATGACGGTAACTACAAGACGCGTGCTGAAGTCAAGTTTGAAGGTGATACCCTTGTTA<br>ATCGTATCGAGTTAAAAGGTATTGATTTTAAAGAAGATGGAAACATTCTCGGACACAAACTCGAGTACAACTA<br>TAACTCACACAATGTATACATCACGGCAGA	Donor DNA sequence including rewritten DNA fragment and two 500 bp homologous arms.

Table S2. Bacterial strains

Strains	Characteristics	Source
E. coli MG1655	*F-lambda-rph-1*	ZOMANBIO
E. coli MG1655BT	MG1655 transformed with the plasmids pUC57-Base64-text and p46Cpf1-OP2	This study
E. coli MG1655BNT	MG1655 transformed with the plasmids pUC57-Base64-newtext and p46Cpf1-OP2	This study
E. coli MG1655HT	MG1655 transformed with the plasmids pUC57-Huffman-text and p46Cpf1-OP2	This study
E. coli MG1655HNT	MG1655 transformed with the plasmids pUC57-Huffman-newtext and p46Cpf1-OP2	This study
E. coli MG1655HC	MG1655 transformed with the plasmids pUC57-Huffman-codebook and p46Cpf1-OP2	This study
E. coli MG1655HNC	MG1655 transformed with the plasmids pUC57-Huffman-newcodebook and p46Cpf1-OP2	This study
E. coli MG1655HI	MG1655 transformed with the plasmids pUC57-Huffman-image and p46Cpf1-OP2	This study
E. coli MG1655HNI	MG1655 transformed with the plasmids pUC57-Huffman-newimage and p46Cpf1-OP2	This study

Table S3. Primer sequences

Name	Nucleotide Sequence (5' to 3')	Comment
Ba-crRNA-F	TAATACGACTCACTATAGAATTTCTACTGTTGTAGAT**acgaagtctgactcgcagcctgtag**	ssDNA templates for *in vitro* crRNA transcription (Base64)
Ba-crRNA-R	**ctacaggctgcgagtcagacttcgt**ATCTACAACAGTAGAAATTCTATAGTGAGTCGTATTA	
Hu-crRNA-F	TAATACGACTCACTATAGAATTTCTACTGTTGTAGAT**tcttctgctggtggttcgttcggta**	ssDNA templates for *in vitro* crRNA transcription (Huffman)
Hu-crRNA-R	**taccgaacgaaccaccagcagaaga**ATCTACAACAGTAGAAATTCTATAGTGAGTCGTATTA	
Ba-rewrite-F	TGGAAAAACGCCAGCAACGC	Primers for the amplification of donor DNA (Base64)
Ba-rewrite-R	TCTGCCGTGATGTATACATTG	
Hu-rewrite-F	CGTTTCGGTGATGACGGTG	Primers for the amplification of donor DNA (Huffman)
Hu-rewrite-R	TCTGCCGTGATGTATACATTG	
Ba-UParm-R	**GCTCTCTAATCATTTAGCTAAGTATCATAGTCTTAAGATGTAACTCGCATTATAGTACTCTCT**AAGCTTATCCCAATGGCGCGCC	Primers for the amplification of donor DNA from the plasmid pUC57-P*tac*GFP (Base64)
Ba-DWarm-F	**CTTAGCTAAATGATTAGAGAGCTAGACTATTCCGCTCTTAAGATTGAACTATGAGAGAGTACTAGATGGCACATTTATATAGTATGC**GAGCTCCGATCCCGCGAAATTAATAC	
Hu-UParm-R	GAATTCATCCCAATACGCGTC	Primers for the amplification of donor DNA from the plasmid pUC57-P*tac*GFP (Huffman)
Hu-DWarm-F	CGATCCCGCGAAATTAATACG	
Hu-text-F	ACGCGTATTGGGATGAATTC**GCCCAGATCCTTTAGAGTCGATCTAGTAGACCG**	Primers for the amplification of donor DNA for text information rewriting (Huffman)
Hu-text-R	GTATTAATTTCGCGGGATCG**CGCATGGCCTGCGATTCCGGTCTACTAGAT**	
Hu-codebook-F	ACGCGTATTGGGATGAATTC**TGTCGTGGGACTCCCAAGTGGTGCGAATCAATTGAATTGGTCTAGGCGATACAAGTTTTGGCTGTCGATGTCGTCTCAAGACTACGACG**	Primers for the amplification of donor DNA for codebook information rewriting (Huffman)
Hu-codebook-R	GTATTAATTTCGCGGGATCG**TCACAAGTCTTGGGAGTCCCACGATTTGCGAGCAATGCCAAAAGTACTAGTCGTCGTAGTCTTGAGACGAC**	
Hu-image-F	TATTGGGATGGAACGAATTC**ACATAGGCGACTCGCCCAATC**	Primers for the amplification of donor DNA for image information rewriting (Huffman)
Hu-image-R	GTATTAATTTCGCGGGATCG**GTCTATAGTATTGGGCCAAATTG**	

REFERENCES AND NOTES

1. Y. Hao, Q. Li, C. Fan, F. Wang, Data storage based on DNA. *Small Struct.* **2**, 2000046 (2021).

2. D. Bennet, T. Vo-Dinh, F. Zenhausern, Current and emerging opportunities in biological medium-based computing and digital data storage. *Nano Select* **3**, 883–902 (2022).

3. K. Matange, J. M. Tuck, A. J. Keung, DNA stability: A central design consideration for DNA data storage systems. *Nat. Commun.* **12**, 1358 (2021).

4. B. H. Nguyen, C. N. Takahashi, G. Gupta, J. A. Smith, R. Rouse, P. Berndt, S. Yekhanin, D. P. Ward, S. D. Ang, P. Garvan, H.-Y. Parker, R. Carlson, D. Carmean, L. Ceze, K. Strauss, Scaling DNA data storage with nanoscale electrode wells. *Sci. Adv.* **7**, eabi6714 (2021).

5. L. C. Meiser, P. L. Antkowiak, J. Koch, W. D. Chen, A. X. Kohll, W. J. Stark, R. Heckel, R. N. Grass, Reading and writing digital data in DNA. *Nat. Protoc.* **15**, 86–101 (2020).

6. J. Koch, S. Gantenbein, K. Masania, W. J. Stark, Y. Erlich, R. N. Grass, A DNA-of-things storage architecture to create materials with embedded memory. *Nat. Biotechnol.* **38**, 39–43 (2020).

7. L. Organick, S. D. Ang, Y. J. Chen, R. Lopez, S. Yekhanin, K. Makarychev, M. Z. Racz, G. Kamath, P. Gopalan, B. Nguyen, C. N. Takahashi, S. Newman, H. Y. Parker, C. Rashtchian, K. Stewart, G. Gupta, R. Carlson, J. Mulligan, D. Carmean, G. Seelig, L. Ceze, K. Strauss, Random access in large-scale DNA data storage. *Nat. Biotechnol.* **36**, 242–248 (2018).

8. L. Organick, Y. J. Chen, S. Dumas Ang, R. Lopez, X. Liu, K. Strauss, L. Ceze, Probing the physical limits of reliable DNA data retrieval. *Nat. Commun.* **11**, 616 (2020).

9. J. L. Banal, T. R. Shepherd, J. Berleant, H. Huang, M. Reyes, C. M. Ackerman, P. C. Blainey, M. Bathe, Random access DNA memory using Boolean search in an archival file storage system. *Nat. Mater.* **20**, 1272–1280 (2021).

10. W. Tang, D. R. Liu, Rewritable multi-event analog recording in bacterial and mammalian cells. *Science* **360**, eaap8992 (2018).

11. H. Lee, D. J. Wiegand, K. Griswold, S. Punthambaker, H. Chun, R. E. Kohman, G. M. Church, Photon-directed multiplexed enzymatic DNA synthesis for molecular digital data storage. *Nat. Commun.* **11**, 5246 (2020).

12. S. S. Yim, R. M. McBee, A. M. Song, Y. Huang, R. U. Sheth, H. H. Wang, Robust direct digital-to-biological data storage in living cells. *Nat. Chem. Biol.* **17**, 246–253 (2021).

13. W. Chen, M. Han, J. Zhou, Q. Ge, P. Wang, X. Zhang, S. Zhu, L. Song, Y. Yuan, An artificial chromosome for data storage. *Natl. Sci. Rev.* **8**, nwab028 (2021).

14. C. Arnold, What's new in clinical CRISPR? *Nat. Med.* **27**, 184–185 (2021).

15. R. Brosh, J. M. Laurent, R. Ordonez, E. Huang, M. S. Hogan, A. M. Hitchcock, L. A. Mitchell, S. Pinglay, J. A. Cadley, R. D. Luther, D. M. Truong, J. D. Boeke, M. T. Maurano, A versatile platform for locus-scale genome rewriting and verification. *Proc. Natl. Acad. Sci. U.S.A.* **118**, e2023952118 (2021).

16. Z. Wu, Y. Zhang, H. Yu, D. Pan, Y. Wang, Y. Wang, F. Li, C. Liu, H. Nan, W. Chen, Q. Ji, Programmed genome editing by a miniature CRISPR-Cas12f nuclease. *Nat. Chem. Biol.* **17**, 1132–1138 (2021).

17. J. Champer, J. Liu, S. Y. Oh, R. Reeves, A. Luthra, N. Oakes, A. G. Clark, P. W. Messer, Reducing resistance allele formation in CRISPR gene drive. *Proc. Natl. Acad. Sci. U.S.A.* **115**, 5522–5527 (2018).

18. A. V. Anzalone, P. B. Randolph, J. R. Davis, A. A. Sousa, L. W. Koblan, J. M. Levy, P. J. Chen, C. Wilson, G. A. Newby, A. Raguram, D. R. Liu, Search-and-replace genome editing without double-strand breaks or donor DNA. *Nature* **576**, 149–157 (2019).

19. M. Naeem, S. Majeed, M. Z. Hoque, I. Ahmad, Latest developed strategies to minimize the off-target effects in CRISPR-Cas-mediated genome editing. *Cell* **9**, 1608 (2020).

20. S. L. Shipman, J. Nivala, J. D. Macklis, G. M. Church, CRISPR–Cas encoding of a digital movie into the genomes of a population of living bacteria. *Nature* **547**, 345–349 (2017).

21. Y. Zhang, L. Kong, F. Wang, B. Li, C. Ma, D. Chen, K. Liu, C. Fan, H. Zhang, Information stored in nanoscale: Encoding data in a single DNA strand with Base64. *Nano Today* **33**, 100871 (2020).

22. Y. Ren, Y. Zhang, Y. Liu, Q. Wu, J. Su, F. Wang, D. Chen, C. Fan, K. Liu, H. Zhang, DNA-based concatenated encoding system for high-reliability and high-density data storage. *Small Methods* **6**, e2101335 (2022).

23. D. Choe, J. H. Lee, M. Yoo, S. Hwang, B. H. Sung, S. Cho, B. Palsson, S. C. Kim, B.-K. Cho, Adaptive laboratory evolution of a genome-reduced Escherichia coli. *Nat. Commun.* **10**, 935 (2019).

24. X. Ao, Y. Yao, T. Li, T. T. Yang, X. Dong, Z. T. Zheng, G. Q. Chen, Q. Wu, Y. Guo, A multiplex genome editing method for Escherichia coli based on CRISPR-Cas12a. *Front. Microbiol.* **9**, 2307 (2018).

25. Y. Ren, Y. Zhang, Y. Liu, Q. Wu, H.-G. Hu, J. Li, C. Fan, D. Chen, K. Liu, H. Zhang, Highly reliable and efficient encoding systems for hexadecimal polypeptide-based data storage. *Fundam. Res.* 10.1016/j.fmre.2021.11.030 (2021).

26. J. S. Chen, E. Ma, L. B. Harrington, M. D. Costa, X. Tian, J. M. Palefsky, J. A. Doudna, CRISPR-Cas12a target binding unleashes indiscriminate single-stranded DNase activity. *Science* **360**, 436–439 (2018).

27. L. Zhang, R. Sun, M. Yang, S. Peng, Y. Cheng, C. Chen, Conformational dynamics and cleavage sites of Cas12a are modulated by complementarity between crRNA and DNA. *iScience* **19**, 492–503 (2019).

28. M. Hao, H. Qiao, Y. Gao, Z. Wang, X. Qiao, X. Chen, H. Qi, A mixed culture of bacterial cells enables an economic DNA storage on a large scale. *Commun. Biol.* **3**, 416 (2020).

29. P. Wang, L. Robert, J. Pelletier, W. L. Dang, F. Taddei, A. Wright, S. Jun, Robust growth of *Escherichia coli*. *Curr. Biol.* **20**, 1099–1103 (2010).

30. G. M. Church, Y. Gao, S. Kosuri, Next-generation digital information storage in DNA. *Science* **337**, 1628 (2012).

31. N. Goldman, P. Bertone, S. Chen, C. Dessimoz, E. M. LeProust, B. Sipos, E. Birney, Towards practical, high-capacity, low-maintenance information storage in synthesized DNA. *Nature* **494**, 77–80 (2013).

32. S. M. Yazdi, Y. Yuan, J. Ma, H. Zhao, O. Milenkovic, A rewritable, random-access DNA-based storage system. *Sci. Rep.* **5**, 14138 (2015).

33. R. N. Grass, R. Heckel, M. Puddu, Robust chemical preservation of digital information on DNA in silica with error-correcting codes. *Angew. Chem. Int. Ed.* **54**, 2552–2555 (2015).

34. J. Bornholt, R. Lopez, D. M. Carmean, L. Ceze, G. Seelig, K. Strauss, paper presented at the Proceedings of the 21st International Conference on Architectural Support for Programming Languages and Operating Systems, Atlanta, GA, USA, April 2016, pp. 2–6.

35. C. Mayer, G. R. McInroy, P. Murat, P. Van Delft, S. Balasubramanian, An epigenetics-inspired DNA-based data storage system. *Angew. Chem. Int. Ed.* **55**, 11144–11148 (2016).

36. R. Lopez, Y. J. Chen, S. Dumas Ang, S. Yekhanin, K. Makarychev, M. Z. Racz, G. Seelig, K. Strauss, L. Ceze, DNA assembly for nanopore data storage readout. *Nat. Commun.* **10**, 2933 (2019).

37. L. Anavy, I. Vaknin, O. Atar, R. Amit, Z. Yakhini, Data storage in DNA with fewer synthesis cycles using composite DNA letters. *Nat. Biotechnol.* **37**, 1229–1236 (2019).

38. H. H. Lee, R. Kalhor, N. Goela, J. Bolot, G. M. Church, Terminator-free template-independent enzymatic DNA synthesis for digital information storage. *Nat. Commun.* **10**, 2383 (2019).

39. Y. Choi, T. Ryu, A. C. Lee, H. Choi, H. Lee, J. Park, S. H. Song, S. Kim, H. Kim, W. Park, S. Kwon, High information capacity DNA-based data storage with augmented encoding characters using degenerate bases. *Sci. Rep.* **9**, 6582 (2019).

40. K. J. Tomek, K. Volkel, E. W. Indermaur, J. M. Tuck, A. J. Keung, Promiscuous molecules for smarter file operations in DNA-based data storage. *Nat. Commun.* **12**, 3518 (2021).

41. C. Xu, B. Ma, Z. Gao, X. Dong, C. Zhao, H. Liu, Electrochemical DNA synthesis and sequencing on a single electrode with scalability for integrated data storage. *Sci. Adv.* **7**, eabk0100 (2021).

42. Y. Chen, Y. Mei, X. Jiang, Universal and high-fidelity DNA single nucleotide polymorphism detection based on a CRISPR/Cas12a biochip. *Chem. Sci.* **12**, 4455–4462 (2021).

43. J. Sun, J. Chen, K. Liu, H. Zeng, Mechanically strong proteinaceous fibers: Engineered fabrication by microfluidics. *Engineering* **7**, 615–623 (2021).

44. N. Kalebic, E. Taverna, S. Tavano, F. K. Wong, D. Suchold, S. Winkler, W. B. Huttner, M. Sarov, CRISPR/Cas9-induced disruption of gene expression in mouse embryonic brain and single neural stem cells in vivo. *EMBO Rep.* **17**, 338–348 (2016).

45. S. L. Shipman, J. Nivala, J. D. Macklis, G. M. Church, Molecular recordings by directed CRISPR spacer acquisition. *Science* **353**, aaf1175 (2016).

46. Y. Mei-Yi, Y. Hai-Qin, R. Gai-Xian, Z. Ju-Ping, G. Xiao-Peng, S. Yi-Cheng, CRISPR-Cas12a-assisted recombineering in bacteria. *Appl. Environ. Microbiol.* **83**, e00947-17 (2017).

47. H. A. De Boer, L. J. Comstock, M. Vasser, The *tac* promoter: A functional hybrid derived from the *trp* and *lac* promoters. *Proc. Natl. Acad. Sci. U.S.A.* **80**, 21–25 (1983).
